# Rare actinobacteria isolated from the hypersaline Ojo de Liebre Lagoon as a source of novel bioactive compounds with biotechnological potential

**DOI:** 10.1099/mic.0.001144

**Published:** 2022-02-25

**Authors:** Andrea Y. Zamora-Quintero, Mónica Torres-Beltrán, Dulce G. Guillén Matus, Irasema Oroz-Parra, Natalie Millán-Aguiñaga

**Affiliations:** ^1^​ Facultad de Ciencias Marinas, Universidad Autónoma de Baja California, Ensenada, Baja California, México; ^2^​ Scripps Institution of Oceanography, University of California, San Diego, La Jolla, California, USA

**Keywords:** actinobacteria, secondary metabolites, anticancer activity

## Abstract

The Ojo de Liebre Lagoon is a Marine Protected Area that lies within a UNESCO World Heritage Site and is a critical habitat for important migratory species such as the grey whale and bird species. Unique hypersaline environments, such as the Ojo de Liebre Lagoon, are underexplored in terms of their bacterial and chemical diversity, representing a potential source for new bioactive compounds with pharmacological properties. Actinobacteria are one of the most diverse and prolific taxonomic bacterial groups in terms of marine bioactive compounds. This study aimed to identify the culturable actinobacterial community inhabiting the Lagoon, as well as to test their potential as new sources of anticancer compounds with pharmacological potential. A selective isolation approach focused on spore-forming bacteria from 40 sediment samples generated a culture collection of 64 strains. The 16S rRNA gene analyses identified three phyla in this study, the Actinobacteria, Firmicutes and Proteobacteria, where the phylum Actinobacteria dominated (57%) the microbial community profiles. Within the Actinobacteria, nine different genera were isolated including the *Actinomadura, Micromonospora, Nocardiopsis*, *Plantactinospora* and *Streptomyces* sp. We observed seasonal differences on actinobacteria recovery. For instance, *Micromonospora* strains were recovered during the four sampling seasons, while *Arthrobacter* and *Pseudokineococcus* were only isolated in February 2018, and *Blastococcus, Rhodococcus* and *Streptomyces* were uniquely isolated in June 2018. Ethyl acetate crude extracts derived from actinobacterial cultures were generated and screened for cytotoxic activity against six cancer cell lines. Strains showed promising low percentages of viability on lung (H1299), cervical (SiHa), colon (Caco-2) and liver (HepG2) cancer lines. Molecular networking results suggest many of the metabolites produced by these strains are unknown and they might harbour novel chemistry. Our results showed the Ojo de Liebre Lagoon is a novel source for isolating diverse marine actinobacteria which produce promising bioactive compounds for potential biotechnological use as anticancer agents.

## Introduction

The Ojo de Liebre Lagoon, Mexico is a hypersaline shallow coastal lagoon (5–12 m deep), located between 27° 35′ and 27° 55′N and 113° 50′ and 114° 20′W, with an extension of 48 km long and 9 km wide. The lagoon is located in a desert area with a very dry (<36 mm rain during winter pluvial season) and warm (22 °C annual average temperature) climate with no fresh water influx into the lagoon. The variations in temperature and salinity in the lagoon are mainly due to the tides, registering salinities of 47 PSU in the internal part [1]. The sediments in the lagoon are mainly fed by the California Current, which reaches the west coast of the peninsula and eventually reaches the lagoon [2]. The Ojo de Liebre Lagoon is a natural reserve that lies within a UNESCO World Heritage Site that sustains numerous ecological interactions and socioeconomic activities. For instance, the lagoon is a key world conservation site, as it is habitat for the reproduction and hibernation of marine mammals (grey whale and harbour seal) and bird species (black brant, red phalarope, and white pelican), and it is also home to endangered species such as different species of sea turtles [3–9]. Additionally, the lagoon supports numerous socio economic activities including the world’s largest solar salt-works (~75000 acres of evaporating salt ponds) [10, 11] that is one of the main economic activities in the region, as well as several ecotourism and fishing activities by the local communities [12–18]. To date, research efforts in the Ojo de Liebre Lagoon have been mostly focused on primary producers and their influence on the natural benthic populations with commercial and economic importance [19–21], leaving a gap in the understanding of the microbial communities’ composition and seasonal dynamics in the lagoon, including the identification and isolation of bacterial phyla with pharmaceutical potential such as the Actinobacteria.

The phylum Actinobacteria is one of the most diverse taxonomic groups and one of the main lineages of the domain Bacteria [22]. Actinobacteria are Gram-positive bacteria that have a high content of guanine and cytosine (G+C) in their genome. They are considered as cosmopolitan free-living microorganisms, which have adapted to a wide range of ecosystems in terrestrial and aquatic environments [23–25], mainly in alkaline soils rich in organic matter [26]. They can adopt different lifestyles ranging from being pathogenic organisms (i.e. some *Corynebacterium *, *Mycobacterium *, *Nocardia* and *Propionibacterium *), soil inhabitants (i.e. *Micromonospora* and *Streptomyces *), plant symbionts (i.e. *Frankia *) to gastrointestinal commensals (i.e. *Bifidobacterium *) [27, 28]. Generally, actinobacteria show growth through a combination of extension of the tip and branching of the hyphae, they produce radial mycelium which facilitates the colonization of substrates far from the centre of growth and some will produce spores as a dispersal and survival strategy [27]. Sporulation may be essential since, being non-mobile bacteria, this is the only way in which they can resist and tolerate the abiotic and/or biotic stress of the habitat [28, 29]. In addition, actinobacteria play an important role in the environment as they degrade organic matter through the production and release of hydrolytic enzymes [27].

Actinobacteria represent one of the most studied and exploited classes of bacteria due to their ability to produce a wide range of biologically active metabolites with industrial, medical, agricultural applications, among others [27, 30, 31]. These are known as secondary metabolites, which are organic molecules that are not directly involved in the development and growth of microorganisms, unlike the primary metabolites that carry out essential physiological functions [32], and that may also be specialized metabolites such as antibiotics, antifungals, antihelminthics, antivirals and immunosuppressives [33]. Studies of actinomycetes as a source of antibiotics began in 1940 when Selman Waksman discovered actinomycin from a culture of *Streptomyces *; since then, actinomycetes have been the main source of antibiotics, deriving approximately two-thirds of all naturally occurring antibiotics in clinical use (e.g. tetracycline, erythromycin, chloramphenicol, vancomycin) [34–36]. Nowadays, actinobacteria are recognized for producing a myriad of compounds for medical use including immunosuppressive agents (rapamycin), anticancer agents (doxorubicin and bleomycin), anthelmintics (avermectin), antifungals (nystatin), and antiviral agents [27, 36, 37], as well as industrially important enzymes, including herbicides (Bialaphos) and insecticides (Spinosad) [38–42].

Currently, actinobacteria isolated from marine environments have received attention due to the structural diversity and unique biological activities of their secondary metabolites. In addition, the use of molecular tools has contributed to expand our view about actinobacteria’s phylogeny allowing us to identify and reclassify actinobacteria into novel taxonomic groups [43]. In the marine ecosystem there is evidence for the occurrence of distinct rare genera of actinobacteria. For instance, new species of actinomycetes isolated from marine sediment have been reported and described, such as *Microbacterium sediminicola* isolated from the mouth of the Samanbula River in Fiji [44]; *Marmoricola aequoreus* from beach sediment on Jeju Island in Korea [45]; *Amycolatopsis marina* isolated from marine sediment of the South China Sea [46]; *Streptomyces qinglanensis* isolated from mangrove sediment collected in China [47]; *Saccharomonospora amisosensis isolated* from the southern Black Sea coast of Turkey [48]; *Salinispora pacifica* isolated from marine sediment from Guam and Palau [49]; *Micromonospora fluostatini* isolated from sediment collected in Thailand [50]; *Nocardioides antarcticus* isolated from Antarctic marine sediment [51]; *Streptomyces otsuchiensis* isolated from marine sediment from Otsuchi Bay in Japan [52]; *Streptomyces marianii* isolated from Indian sediment [53].

Thus, current research efforts are focused on finding novel actinobacteria genera from marine environments and discovering the myriad of novel therapeutic drugs, which may be effective at combating a range of life-threatening diseases such as cancer. These novel genera, also referred to as ‘rare’, have accounted for approximately 100 bioactive compounds from 2007 to 2013. Among the most prolific rare genera are *Salinispora* (20 new compounds), *Verrucosispora* (18 new compounds), *Nocardiopsis* (12 new compounds), *Actinoalloteichus* (11 new compounds), *Marinispora* (ten new compounds) and *Micromonospora* (nine new compounds). Currently, four of these compounds are found in clinical trials against cancer, anthrax and anti-inflammatory drugs, of which three were obtained from the genus *Salinispora* [54]. For instance, pure active compounds extracted from *Salinispora tropica* have shown inhibitory effects in many malignant cell types [55]. In particular, Salinosporamide A, is a novel rare bicyclic beta-lactone gamma-lactam isolated from *S. tropica* [56, 57] that is currently being evaluated in multiple phase I trials for solid tumours, lymphoma and multiple myeloma. As cancer still remains one of the most serious human health problems, there is an ongoing need to find bioactive compounds derived from marine actinobacteria to be used as anticancer agents.

The Ojo de Liebre Lagoon is a unique ecosystem where physicochemical and seasonal dynamics may play a role in shaping the microbial communities inhabiting the sediments, including the diversity and distribution of marine actinobacteria. Therefore, in this study we aimed (1) to generate novel knowledge on the composition of the culturable actinobacteria inhabiting the Ojo de Liebre Lagoon, focusing primarily on their isolation and phylogenetic diversity, (2) to identify patterns of distribution for the bacteria isolated, focusing primarily on changes in actinobacteria recovery among sampling seasons and (3) to identify actinobacterial strains producing bioactive compounds with anticancer potential. Our results contribute to filling the gap about the Ojo de Liebre culturable microbial communities, and provide unprecedented knowledge on actinobacteria from hypersaline marine environments supporting their role as a source of bioactive compounds with anticancer potential.

## Methods

### Marine sediment collection

**Fig. 1.**
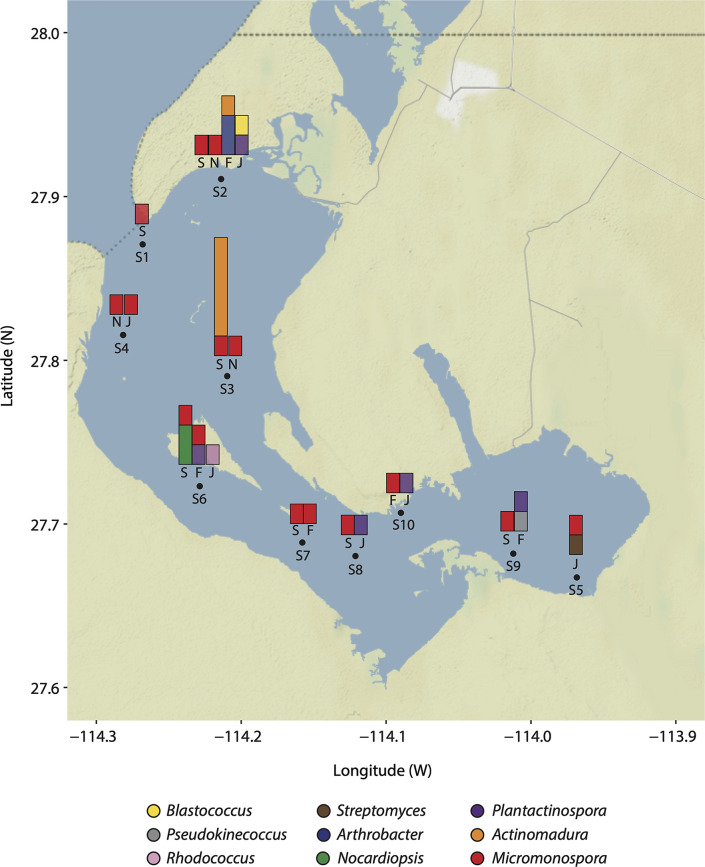
The Ojo de Liebre Lagoon. Sampling stations are shown in the map as numbered black dots (**S1-S10**) distributed along the Lagoon. Bar charts on top of sampling stations depict the seasonal distribution (September 2017 = S, November 2017 = N, February 2018 = F and June 2018 = J) and abundance of isolated actinobacteria genera. Bars for sampling seasons in which no actinobacteria were isolated are not shown. Bars are coloured according to key.

Four seasonal samplings were carried out (September 2017, November 2017, February 2018 and June 2018) in the Ojo de Liebre Lagoon to collect marine sediment samples at ten different sites with a maximum depth of approximately 13 metres (Fig. 1). A total of 40 sediment samples were collected manually by freediving. Sediments were scooped into sterile Whirl-Pak bags and brought up to the surface. Sediment samples were preserved on ice immediately after collection for their later processing and analysis in the laboratory. Along with sediment samples collection, temperature and salinity profiles were measured at each site using a CTD YSI CastAway in order to obtain environmental conditions information from the sampling site (Table S1).

### Processing marine sediment samples

The 40 sediment samples were pretreated using the previously described stamping method used by Mincer *et al.* (2002) [58] with the modifications used by Millán-Aguiñaga *et al.* (2019) [59]. Briefly, dried sediment was inoculated onto seven different agar media by stamping in a circular manner, giving a serial dilution effect to target the isolation of spore-forming bacteria only, as this is an important characteristic for identifying and isolating actinobacterial strains. Most media were supplemented with either artificial Instant Ocean sea salt mix or natural sea salt from the Ojo de Liebre Lagoon. The compositions of each media was as follows: *Oats* [commercial oats, 14 gl^−1^; agar, 14 gl^−1^; pH 7]; *10 %* A1 [starch, 1 gl^−1^; yeast 0.4 gl^−1^; peptone, 0.2 gl^−1^; agar, 14 gl^−1^, sea salt, 22 gl^−1^; pH 7]; *10 %* A1-35 [starch, 1 gl^−1^; yeast 0.4 gl^−1^; peptone, 0.2 gl^−1^; agar, 14 gl^−1^, sea salt, 35 gl^−1^; pH 10.15]; *SW* [agar, 14 gl^−1^, sea salt, 22 gl^−1^]; *SW-35* [agar, 14 gl^−1^, sea salt, 35 gl^−1^; pH 10.15]; *SW-LOL-35* [agar, 14 gl^−1^; lagoon sea salt, 35 gl^−1^; pH 10.15]; *SW-LOL-22* [agar, 14 gl^−1^, lagoon sea salt, 22 gl^−1^; pH 7]. All inoculated petri dishes were incubated at room temperature (~20 °C) and colony growth was monitored every week for a period of 6 months. The strains isolated were subcultured until pure using A1 media [starch, 10 gl^−1^; yeast 4 gl^−1^; peptone, 2 gl^−1^; agar, 14 gl^−1^, sea salt, 22 gl^−1^; pH 7]; cryopreserved with glycerol (10%) and stored at −20 °C.

### 16S rRNA gene amplification and phylogenetic analysis

Isolated strains were cultured in medium A1 with shaking at 150 r.p.m. for 7 days. For the genomic DNA (gDNA) extraction, 1 ml culture was withdrawn and centrifuged at 10 000 r.p.m. for 10 min. Once the bacterial pellet was formed, supernatant was removed and the DNA was extracted according to the Omni Bacteria DNA purification protocol (Omni International). Extracted gDNA was used to amplify the 16S rRNA gene. Polymerase chain reaction (PCR) was performed using the universal primers: FC27 and RC1492. The PCR programme conditions were 94 °C for 10 min, 35 cycles of 94 °C for 1 min, 63 °C for 1 min and 72 °C for 1 min; followed by 7 min at 72 °C. Amplification quality was determined by visualization on 0.8 % agarose gel run (30 min/110V). Samples showing an expected band of approximately 1400 bp were sent to ETON Bioscience Inc. of San Diego, California for sequencing using the Sanger sequencing platform. The 16S rRNA sequences were visualized and trimmed using the Geneious 2021.1.1 programme for subsequent similarity analysis using the EZTAXON database (https://www.ezbiocloud.net/). Taxonomic assignment of bacterial strains was done based on the highest percentage of similarity (>98.6 %) among the reference and query trimmed sequence. Maximum-Likelihood (ML) and Neighbour-Joining (NJ) trees were created using the Geneious 2021.1.1 programme; using only the strains with 16S rRNA sequence size greater than 1100 bp. Analyses included 1000 bootstrap replicates using the most complex model GRT+GAMMA.

### Fermentation and metabolite extraction

Twelve strains were selected based on their taxonomic affiliation for cell viability assays and metabolite analysis. Strains affiliated with the *Actinomadura, Micromonospora* and *Nocarcardiopsis* were prioritized for analysis since they have shown potential for novel secondary metabolites production. The *Micromonospora* strains were also prioritized to include strains from different seasons and sampling sites. Selected strains were pre-cultured (25 ml, 28 °C, 150 r.p.m. for 9 days) in A1 and then transferred to 100 ml culture for 9 days (28 °C, 150 r.p.m.). Ethyl acetate was added to the cultures, shaken for 3 h (150 r.p.m., 28 °C,) and then a liquid-liquid extraction was performed [60]. The organic phase was then dried *in vacuo* and weighed.

### Cell viability assay on cancer cell lines

Human cancer cell lines including lung (H1299), cervical (HeLa, SiHa and CaSki), colon (Caco-2), and liver (Hep G2), were used to conduct cell viability assays. Cancer cell lines were cultured in RMPI-1640 and EMEM medium, both supplemented with 10 % of foetal bovine serum (FBS, Sigma-Aldrich) and 1 % antimycotic antibiotic (Sigma-Aldrich). Cells were incubated at 37 °C with 5 % CO_2_. Cell viability assay was evaluated by the cytotoxic colorimetric reaction CellTiter 96 Aqueous One Solution Proliferation Assay Kit (MTS) (Promega). The MTS reaction is carried out by enzymes in living cells that convert tetrazolium salt into a soluble formazan product that is coloured. For each cancer cell line, a total of 5×10^4^ cells per well were added to culture plates and were incubated during 24 h at 37 °C with 5 % CO_2_. After the incubation period, 50 µg of each crude extract were added to culture plates in triplicate, and 1 % DMSO (vehicle) was used as negative control (C-) and 50 µg of etoposide was used as positive control (C+). Plate was incubated for 24 h, followed by an addition of 20 µl MTS to each treatment, and absorbance was measured at 490 nm, using a microplate reader EPOCH (Biotek, Winooski, United States), in order to determine the number of viable cells. All results were normalized to C-.

### Mass spectrometry

Resulting crude extracts were analysed by Liquid Chromatography (Agilent Technologies 1200 series Liquid Chromatograph) in tandem with an ESI Ion Trap Mass spectrophotometer (Bruker Daltonics). Liquid chromatography separation was performed in a reverse-phase Kinetex C_18_ column (pore size: 5 µm, particle size 100 Å dimensions: 150 mm × 4.6 mm) at a flow rate of 0.750 µl m^−1^, with an elution gradient of 5–100% Acetonitrile with 0.1 % formic acid in water over 18 min. Mass spectrometry acquisition was in the positive ion mode with a collision-induced energy of 30 % and mass range of 100–2200 Da; scan speed of 32 500 m/z/sec and capillary of 4500 V; with smart ion charge control (ICC) of 20 000, and maximum accumulation time of 100 ms; with a mass acquisition error of about 0.5 m/z. The MS/MS was set on AutoMS_2_ with an average of three experiments with two precursor ions, and an active exclusion after two spectra or 1 min, using collision-induced dissociation (CID). Nitrogen was used as nebulizing gas (45 psi) and drying gas (8 l min^−1^, 350 °C). The raw LC-MS data were converted to mzXML format by CompassXport software for further analysis.

### Molecular network

We pre-processed the mass spectrometry data using MZMINE 2.53 [61] to remove baseline noise. Noise levels for MS1 and MS2 were set at 1.0E5 and 1.0E3, respectively and we selected centroid as the method for mass detection and the ADAP module as chromatogram builder [62]. All resulting data containing MS/MS fragmentation were exported and analysed in GNPS [63] using Feature Based Molecular Networking (FBMN) [64]. We used the following network parameters, the precursor ion mass tolerance was set to 2.0 Da and to 0.5 Da for fragment ion tolerance. The molecular network was created from parent ions sharing a cosine score above 0.7 and more than six matched peaks. Library matches were required to have a score above 0.75 and at least four matched peaks. The MolNetEnhancer workflow [65] was used to inform structural annotations further within the molecular network; the tool utilizes CalssyFire chemical ontology for the chemical class annotations. For further visualization and network manipulation we used the software Cytoscape [66]. The edge width was scaled based on the cosine similarity score and the node size was based on the most sensitive cell line observed from the cell viability assays.

## Results

### Culturable bacteria distribution and taxonomic diversity

To first investigate the diversity of actinobacteria using culture-dependent methods, a total of 40 marine sediment samples were collected from the Ojo de Liebre Lagoon (ten sediment samples for each sampling month corresponding to September 2017, November 2017, February 2018 and June 2018). Sampling sites (S1 to S10) were located across the lagoon, where stations S1, S2 and S4 are located by the mouth of the lagoon, stations S3, S6-S8 and S10 are distributed in the centre, and stations S5 and S9 at the head of the lagoon (Fig. 1).

A total of 64 bacterial isolates were obtained using a selective isolation method based on morphology (Fig. S1, available in the online version of this article). The recovery of bacterial isolates showed a relation with the sampling site and the culture media used. For instance, sampling stations S2 and S3 showed the highest abundance (17 %) of the total isolated bacteria, while stations S8 and S10 showed the lowest abundance (5 %) (Fig. S2a). Regarding culture media, we observed a greater abundance (27 %) of strains isolated from medium 10% A1 and a lower abundance (3 %) from the SW-LOL-35 gr medium (Fig. S2b). In addition, we observed that the recovery of isolated strains was distributed among culture media for different sampling locations. For stations S2 and S3 the total number of isolates were obtained from five different culture media, while in stations S8 and S10 isolates were obtained from only two culture media (Fig. S2c).

**Fig. 2.**
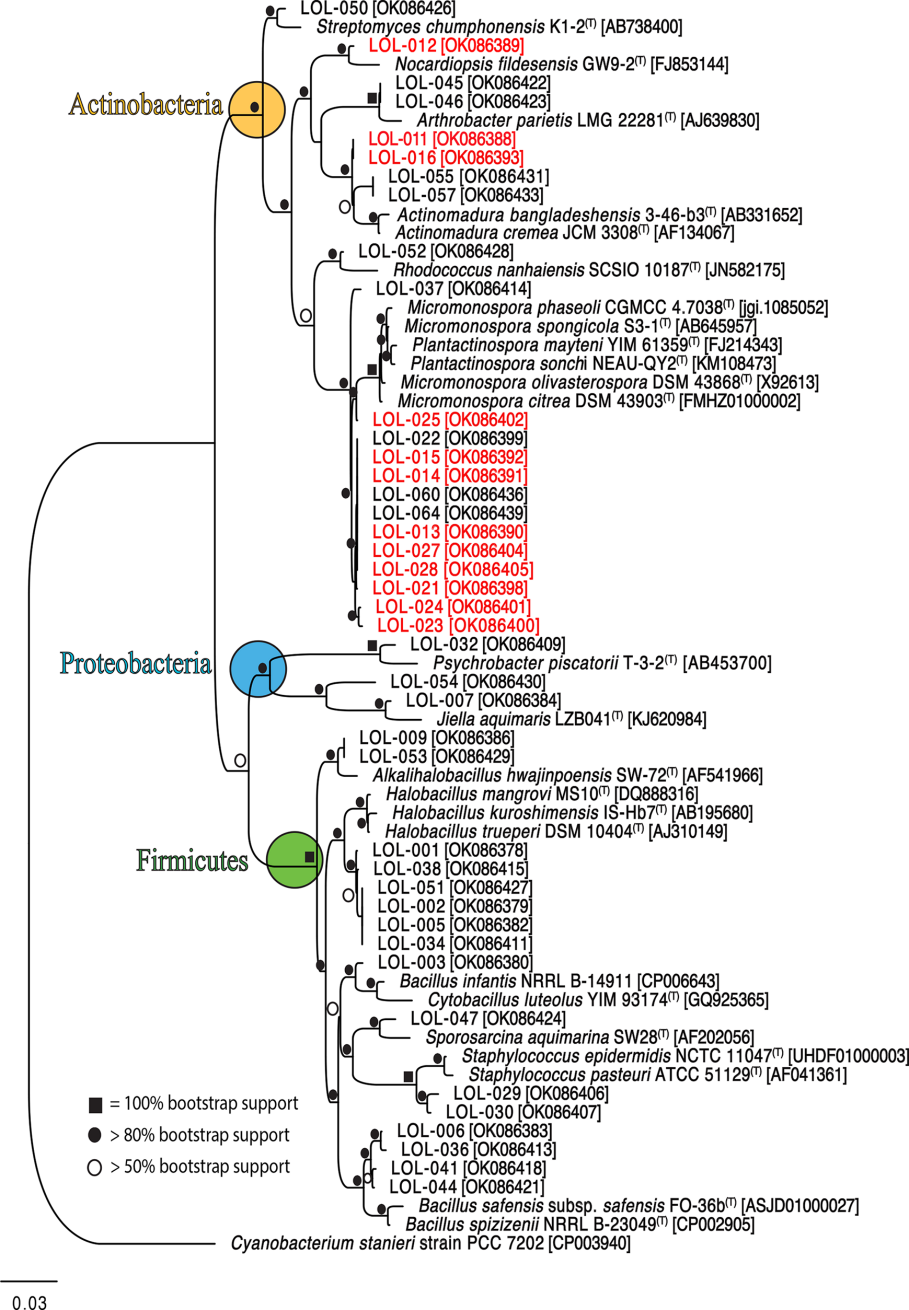
Maximum likelihood tree based on 16S rRNA gene sequences of 41 strains isolated from Ojo de Liebre lagoon sediments. Red colour-coded strains indicate that they were selected for chemical extraction analysis. The tree included the closest type strains, accession numbers and bootstrap values higher than 50 %. Different phyla are represented in different circles; yellow: Actinobacteria, blue: Proteobacteria and green: Firmicutes.

Taxonomic affiliation of isolated strains was conducted by comparing 16S rRNA sequences from isolates with reference strains. A total of 62 strains (97 % of the total number of isolates) were taxonomically identified within three phyla and seven different genera (Table S2), while the rest (3%) remained unclassified due DNA extraction quality. The three phyla identified in this study were the Actinobacteria, Firmicutes and Proteobacteria (Fig. 2). The 16S rRNA gene sequences (~1186 bp length) from 41 strains were used to construct the ML phylogenetic tree. The 16S rRNA sequences with less than 750 bp were not included in the alignment (Fig. 2). The lowest 16S rRNA similarity within the Actinobacteria clade was observed from two *Actinomadura* strains (LOL-055 and LOL-057) sharing 99.4 % similarity with the closest type strain *Actinomadura livida *. A phylogenetic tree including 40 sequence types from *Actinomadura* described species (Fig. S5) verified the supported clade of these two strains (Fig. S3). Within the *Micromonospora* group, LOL-strains were cladding separately from the closest type strains (Fig. 2). The presence of five different clades from the *Micromonospora* LOL-strains was confirmed using 51 *Micromonospora* described species (Fig. S4). All the clades mentioned above were well supported, suggesting future efforts should focus on using more comprehensive approaches i.e. full genome sequencing and multi-locus analysis to differentiate potential novel species within the genus *Actinomadura* and *Micromonospora *.

Regarding phyla distribution and abundance, the Actinobacteria phylum was the most abundant (57 %), followed by the Firmicutes (36 %) and the lowest in abundance (7 %) was the Proteobacteria phylum (Fig. S2a). The phylum Actinobacteria showed the greatest abundance having 34 strains present across all sampling stations (Fig. S2a), except for station S5 where the Firmicutes showed a greater abundance. In fact, the genera *Halobacillus* and *Bacillus* sp. within the Firmicutes showed the largest distribution as it was isolated from eight sampling stations (Fig. S5a). In contrast, the Proteobacteria phylum was only isolated in stations S2, S4 and S8, with four strains in total (Fig. S2a). Furthermore, we observed taxonomic diversity followed a similar pattern to that of the abundance across sampling stations. For instance, in stations with high abundance values, such as S2, 11 strains were isolated and these were affiliated with eight different genera (Fig. S5a). In comparison, in stations showing the lowest abundance values, such as S8 and S10, only three different genera were observed (Fig. S5a). In addition, we observed that medium 10 % A1 was highly effective for bacterial growth and recovery as 15 strains belonging to the three phyla were isolated. However, the highest recovery of Actinobacteria strains was observed in SW medium from which nine strains were isolated. In contrast, from medium SW-LOL-35 gr only two strains were isolated (Fig S5b).

Moreover, we observed seasonal differences in the recovery of strains affiliated with the identified phyla across sampling stations. Although stations S2 and S4 showed the greatest diversity of isolated strains, with the three phyla being present over the sampling period, we identified presence patterns along the lagoon throughout sampling seasons. For instance, we isolated bacteria affiliated with the Actinobacteria, Firmicutes and Proteobacteria from nine stations in samples collected in September 2017. Overall, during this month the Actinobacteria showed the largest distribution as these were isolated from seven sampling stations (Fig. S6a), and particularly from station S1 (lagoon’s mouth) only Actinobacteria strains were isolated (Fig. 1 and S6a). In comparison, from station S5 (lagoon’s head), only Firmicutes strains were isolated (Fig. S6a). In November 2017, we were able to isolate bacteria only from seven sampling stations, and we observed Firmicutes showed a greater distribution along stations and were isolated from stations located at the mouth and head of the lagoon. In contrast, Actinobacteria was only recovered from three stations (S2, S3 and S4) (Figs 1 and S6b). In February 2018, we were able to isolate bacteria from six sampling stations, and we observed Actinobacteria being the one with the greatest distribution as strains were isolated from five stations (Figs 1 and S6c). In this season, Firmicutes were only isolated from samples collected at the head of the lagoon, while Proteobacteria strains were completely absent during this season (Fig. S6c). During the last sampling season, June 2018, we isolated bacteria from eight stations of which in six stations we have representatives of the phylum Actinobacteria (Fig. 1 and S6d). In this season the presence of Proteobacteria is observed in a sampling station. Both in the mouth and in the head of the lagoon the presence of Firmicutes is observed. In this season, actinobacteria were isolated from the head of the lagoon (Fig. S6d).

### Actinobacteria diversity in the Ojo de Liebre Lagoon

A total of 34 Actinobacteria strains affiliated with nine genera were identified, including *Actinomadura, Arthrobacter*, *Blastococcus *, *Micromonospora, Nocardiopsis*, *Plantactinospora, Pseudokineococcus*, *Rhodococcus* and *Streptomyces* (Fig. S5b). The genus *Micromonospora* showed the highest abundance with 14 strains isolated, while from the genera *Blastococcus *, *Pseudokineococcus *, *Rhodococcus *, and *Streptomyces* only one representative strain was isolated (Fig. S5b).

Actinobacteria strains were particularly isolated from medium SW, which showed the highest number of recovered strains (nine in total) (Fig. S5b). However, medium SW did not show the highest diversity of recovered strains, since only three different genera were isolated, including the *Actinomadura *, *Nocardiopsis and Micromonospora*. In comparison, the oat medium showed the highest diversity with six different genera isolated, including *Actinomadura, Arthrobacter, Micromonospora, Plantactinospora, Pseudokineococcus,* and *Streptomyces *. Additionally, the *Micromonospora* was the most recovered genus as representative strains were isolated from all culture media used (Fig. S5b).

The spatial and seasonal distribution and abundance of Actinobacteria was also variable. Generally, *Micromonospora* strains were observed in all stations during the four sampling seasons. The station with the highest diversity and abundance of Actinobacteria strains was S2, with seven strains represented in five different genera: *Actinomadura, Arthrobacter, Blastococcus*, *Micromonospora* and *Plantactinospora *. In contrast, the station with the lowest abundance was S1, with only one *Micromonospora* strain isolated (Fig. 1). Regarding the abundance of Actinobacteria genera recovered by sampling season, in September 2017 we observed the highest abundance (14 total) of recovered strains (Fig. 1), while in November 2017 we observed the lowest abundance with only three strains recovered from all stations. In addition, we observed changes in the taxonomic diversity of isolated strains associated with the sampling season. For instance, during February and June 2018, we observed the highest diversity of Actinobacteria recovered, with five different genera isolated across sampling stations, including *Actinomadura *, *Arthrobacter *, *Blastococcus, Plantactinospora, Pseudokineococcus, Rhodococcus* and *Streptomyces* (Fig. 1). Of note, *Arthrobacter* and *Pseudokineococcus* were only isolated in February 2018, while *Blastococcus, Rhodococcus* and *Streptomyces* were uniquely isolated in June 2018 (Fig. 1).

### Cell viability assay

**Fig. 3.**
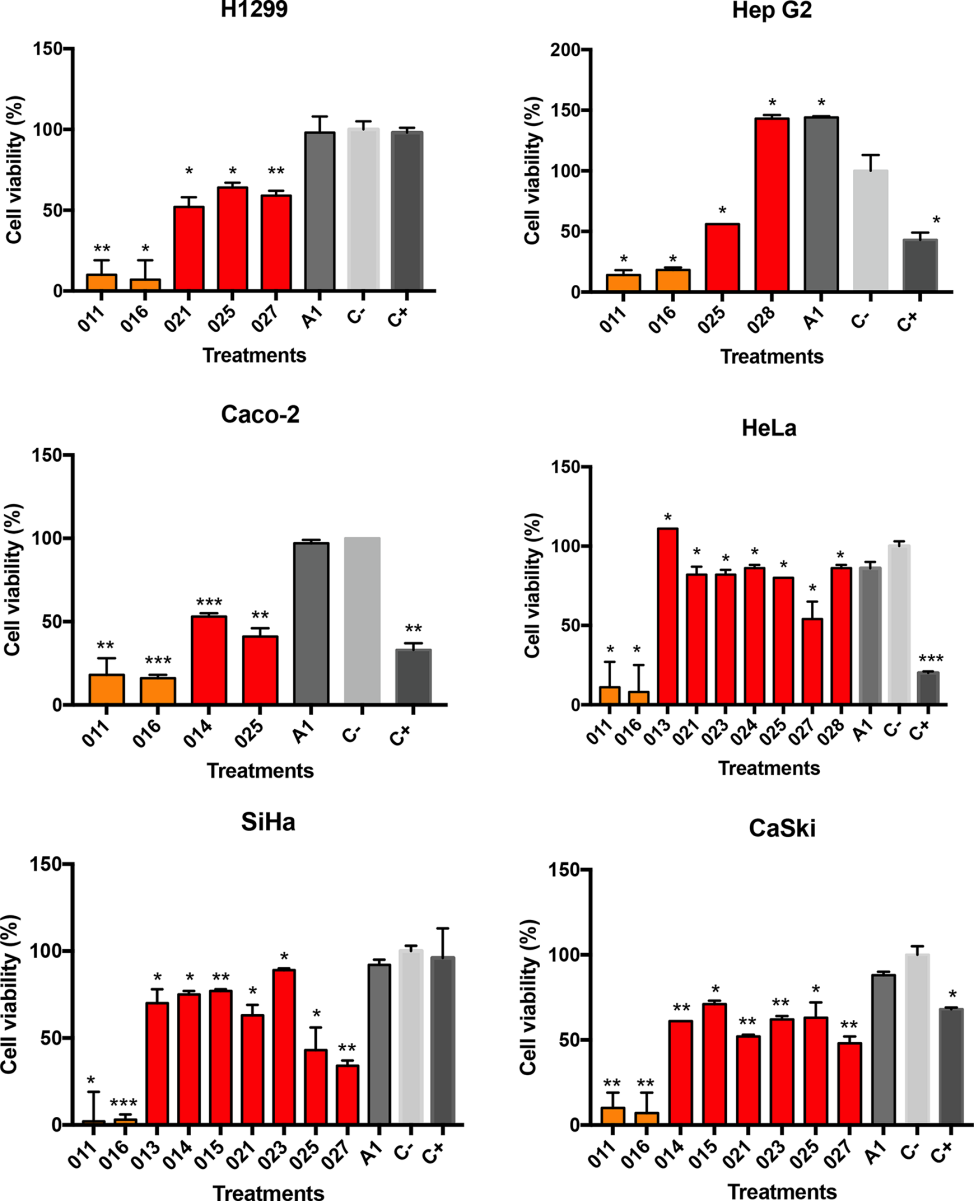
Cytotoxic assays of actinobacteria crude extracts against human cancer cell lines including lung (H1299), cervical (HeLa, SiHa and CaSki), colon (Caco-2), and liver (Hep G2). Cells were treated with 50 µg of each extract. Cell viability was evaluated using MTS assay by measuring absorbance at 490 nm. Then 50 µg of etoposide was used as C+ and 1 % DMSO as a C- (vehicle). A1 means for bacteria culture medium to show no false positive in the cell viability reduction. Results were expressed as mean+/-SEM. P*<0.05, P**<0.01 and P***<0.001 vs. C-. Unpaired *t*-test was used for statistical analysis. Bars are colored based on actinobacterial genus (Actinomadura = orange and Micromonospora = red), while culture medium and controls remained in grayscale.

To initially evaluate the metabolites produced by selected strains for potential anticancer activity, the cytotoxic effect of crude extracts was evaluated by colorimetric MTS reactive assay [67]. It was possible to test only 11 of the 12 crude extracts obtained as the one from strain LOL-012 could not be resuspended to reach the minimum concentration for assays. In almost all assays positive control (C+) showed a significant difference (P*<0.05, P**<0.01, P***<0.001) on decreased cell viability compared to negative control (C-). Regarding the crude extracts activity, we observed a significant decrease in cell viability after 24 h treatments with crude extracts obtained from *Micromonospora* and *Actinomadura* strains (Table S3). Overall, crude extracts from *Micromonospora* strains showed cell proliferation decreased for all cancer lines tested, particularly those from LOL-025 and LOL-027 (Fig. 3). We observed cell viability significantly decreased compared with negative control showing minimum values of cell proliferation as follows, for HeLa we observed cell viability decreased up to 54 % (P*<05), for SiHa we observed cell viability decreased up to 34 % (P**<0.01), and for CaSki we observed cell viability decreased up to 48 % (P**<0.01) (Fig. 3). Regarding the H1299, HepG2 and Caco-2 cell viability decreased up to 66 % (P**<0.01), 56 % (P*<05) and 41 % (P**<0.01) (Fig. 3). In addition, crude extracts from *Actinomadura* strains (LOL-011 and LOL-016) showed the lowest cell viability values for all cancer lines tested (Fig. 3, Table S3). Crude extract from strain LOL-011 significantly decreased cell viability for H1299 (8%, P**<0.01), HepG2 (14 %, P*<05), Caco-2 (18 %, P**<0.01), HeLa (11 %, P*<05), SiHa (2 %, P*<05) and CaSki (10 %, P**<0.01) (Fig. 3), and crude extract from strain LOL-016 significantly decreased cell viability for H1299 (4 %, P*<05), HepG2 (18 %, P*<05), Caco-2 (16%, P***<0.001), HeLa (8 %, P*<05), SiHa (3 %, P***<0.001) and CaSki (7 %, P**<0.01) (Fig. 3).

### Secondary metabolites potential

**Fig. 4.**
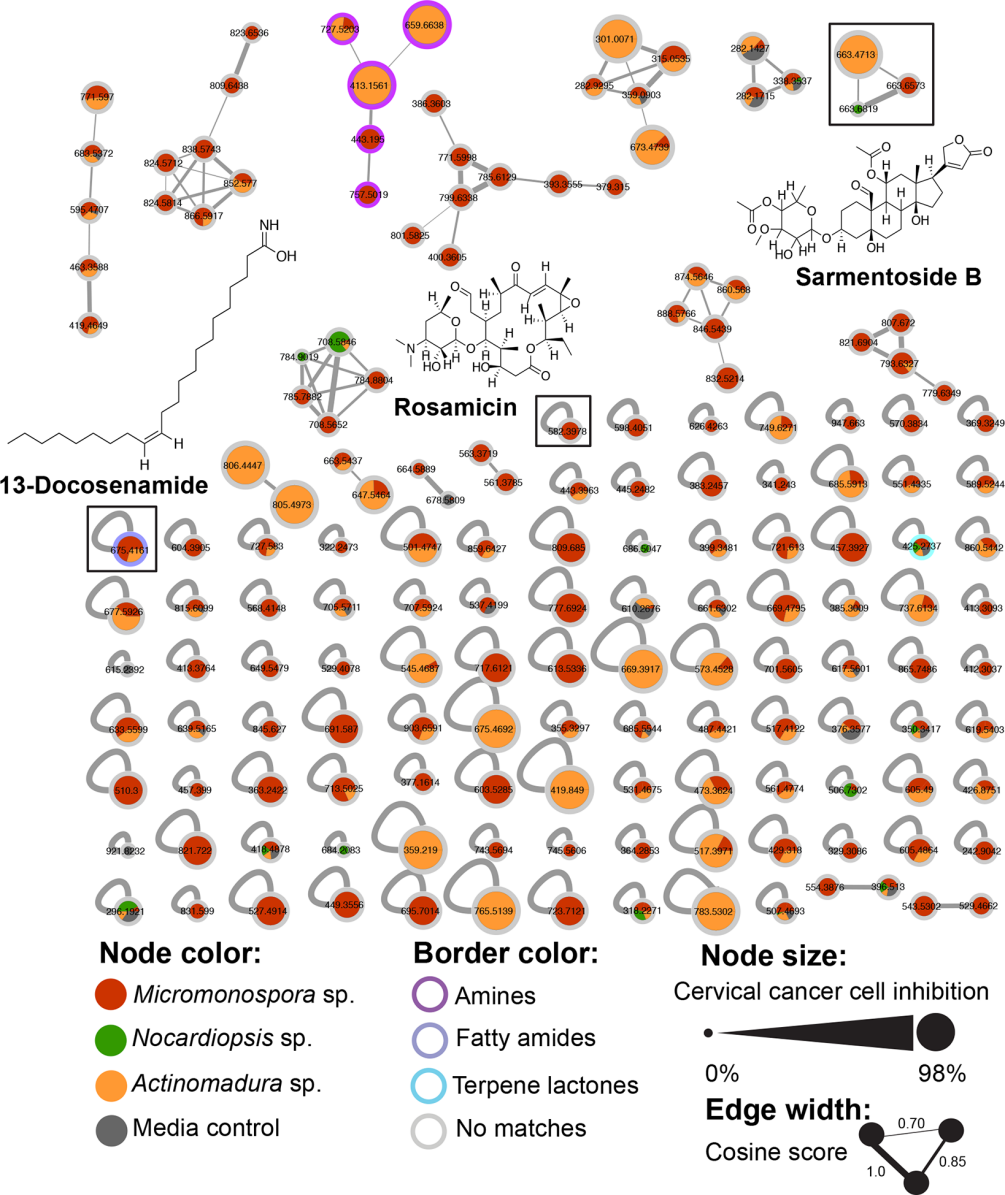
Molecular network of 12 crude extracts. Nodes are colour-coded based on bacterial genus, grey represents parent ions that are found in the culture media control. The node outer rings represent the chemical class annotation by MoltNetEnhancer; parent ions without matches are coloured grey. Metabolites identified via GNPS library search are shown indicating their placement in the network. Node size represents SiHa cervical cancer cell inhibition percentage; which showed the highest sensitivity among the cell lines tested with crude extracts. Edge width is based on cosine score values with wider edges connecting more similar nodes.

Twelve strains taxonomically affiliated with the *Actinomadura, Micromonospora,* and *Nocardiopsis* were prioritized for metabolomic analysis since members of these genera can be prolific producers of novel specialized metabolites. From the 12 crude extracts we constructed a molecular network containing 164 nodes, including 102 singletons and ten molecular families, representing 37.8 % of the total nodes in the network; with a mode size of five nodes, ranging from eight to two nodes per molecular family. *Micromonospora* strains produced 40 % of the ions, *Actinomadura* 8.5 %, and *Nocardiopsis* only 5.5 %. In total, 32 % were shared nodes across all strains, and the remaining 13.5 % nodes were also present in the media control. Through the GNPS library search we found matches to three different compounds: the macrolide antibiotic Rosamicin originally isolated from *Micromonospora rosaria,* but found in other *Micromonospora* strains as well [68], the glycoside Sarmentoside B, originally reported from the plant *Strophanthus sarmentosus* [69] and the fatty amide 13-Docosenamide [70]. We did not find matches to *Actinomadura* or *Nocardiopsis* known metabolites. To acquire further structural information, the network was annotated using the *in silico* tool MolnetEnhancer [65] allowing us to identify three different chemical classes of functional groups including amines, fatty amides and terpene lactones. However, the majority of nodes remained unclassified. From the ten molecular families we observed, nine are unidentified but one is annotated as Amine-related from the MolnetEnhancer analysis (Fig. 4).

Half of the molecular families mostly included nodes associated with *Micromonospora* strains with few others shared among *Actinomadura* sp. and *Nocardiopsis* sp. We identified one genus-specific molecular family from four *Micromonospora* strains: LOL-014, LOL-015, LOL-021, and LOL-027. After comparing their LC-MS/MS chromatograms, we observed two shared parent ions, 771 m/z and 785 mz, which did not match any known *Micromonospora* metabolites from the GNPS library nor the Natural Products Atlas [71] (Fig. S7). In the case of *Actinomadura *, we observed one genus-specific molecular family from two *Actinomadura* strains, LOL-011 and LOL-016, with only two nodes with a mass of 805 m/z and 806 m/z ([M+Na]^+^), that we hypothesize might be different ions from the same metabolite, or analogues, because they share the same retention time and come out in a wider peak (Fig. S8) with no matches to the GNPS library. However, in the Natural Products Atlas, the antibiotic GE23077 B1/B2 is listed as a metabolite from *Actinomadura* with a parent ion [M+H]^+^ of 806.3527 m/z. Nonetheless, our mass data comes from a low accuracy mass spectrometer and we could not calculate a molecular formula in order to annotate these ions in relation to GE23077 B1/B2. Molecular networking is based on fragmentation patterns on top of ion masses, thus the fact that the majority of the produced ions did not match with any chemical classification might suggest many of the metabolites produced by these strains are unknown and they might harbour novel chemistry.

## Discussion

In our study we focused on understanding the distribution and diversity of marine actinobacteria in the Ojo de Liebre Lagoon, and tested their potential metabolism for the production of bioactive compounds. Our study combined culture-dependent methods for strain isolation and their further taxonomic affiliation based on 16S rRNA gene sequencing and phylogenetic analysis. Although selective, our identification methods proved to be effective for the isolation and identification of potentially halophilic bacteria and rare marine actinobacteria which may harbour novel metabolisms for the production of bioactive compounds. We explored the bioactive potential of actinobacterial strains using a molecular networking approach and cytotoxic activity, observing promising results for anticancer activity. Combined, our results showed marine environments with extreme physicochemical conditions, such as the Ojo de Liebre Lagoon, may harbour a high diversity of rare actinobacteria genera with potential metabolism for novel bioactive compounds.

Throughout sampling stations and seasons, the distribution and abundance of the bacterial phyla identified in the Ojo de Liebre Lagoon were variable. It has been reported that changes in environmental factors such as temperature, salinity and nutrient availability may have an effect on shaping microbial communities’ composition [72]. The Ojo de Liebre Lagoon is a marine system where its mouth has greater interaction with marine currents compared to the head, and consequently a gradient in temperature, salinity and nutrients occur along the lagoon [73]. When comparing physicochemical conditions of two most representative stations from mouth (S1) and the head (S5), station S1 showed an average temperature of 20.26 °C and an average salinity of 34.4 PSU, while station S5 an average temperature of 21.09 °C and an average salinity of 42.4 PSU (Table S1). Phyla distribution may be associated with physicochemical conditions in the lagoon, in particular with high salinity concentrations. For instance, we observed a greatest recovery of strains affiliated with Firmicutes and Actinobacteria in station S5, in comparison with S1, suggesting the strains isolated from this station may show higher tolerance for high salinity concentrations. Because hypersaline environments are generally considered as hostile environments, the diversity of halophilic microorganisms has been little studied [74]. Nonetheless, the most abundant microorganisms in these environments are commonly phototrophic i.e. Cyanobacteria, anaerobic phototrophic, anaerobic Gram-negative bacteria and Gram-positive bacteria such as *Halobacillus *, *Bacillus* within the Firmicutes, and some Actinobacteria [75–84]. In this study, we observed bacteria affiliated with *Halobacillus* (12 % of the isolated strains) and *Bacillus* (20 % of the isolated strains) were the two most abundant genera observed and were isolated from station S5 (Fig. S5a). Furthermore, 63 % of the strains belonging to the genus *Bacillus* and 37 % of the *Halobacillus* strains were isolated from the medium 10 % A1-35 gr, which was the medium with the highest salt concentration (35 gr), supporting Firmicutes resistance for high salinity environmental conditions (Fig. S2c).

Regarding actinobacteria isolated from station S5, we isolated those affiliated with the *Micromonospora* and *Streptomyces* (Fig. 1). Occurrence of actinobacteria affiliated with *Micromonospora and Streptomyces* in hypersaline environments such as marine salters have been reported [85, 86], supporting the tolerance of these genera for high salinity environmental conditions as those observed in Laguna Ojo de Liebre. Regarding the diversity and abundance of Actinobacteria, these varied with the sampling season (Fig. 1). Overall, we observed the highest Actinobacteria diversity during February and June 2018, corresponding to the sampling period after the whale season. The grey whale season at Ojo de Liebre begins in December and ends in mid-April. During their stay, grey whales feed on invertebrates found in the bottom, resuspending the marine sediments [87]. This process may mix water column and sediment microbial community members, and likely have an impact on the isolated strains' diversity. In comparison, during September 2017 we observed the highest abundance of Actinobacteria strains, corresponding to higher temperatures registered in the lagoon (up to 23.8 °C) in comparison to other seasons (Table S1). Actinobacteria have been reported to be generally more abundant during the warmer seasons in the most superficial layers of sediment (0.20 cm) [88].

In this study, 34 strains of actinobacteria were isolated using seven culture media (Fig. S5b) and represented nine genera (*Actinomadura, Arthrobacter, Blastococcus, Micromonospora, Nocardiopsis, Plantactinospora, Pseudokineococcus, Rhodococcus* and *Streptomyces *). Our isolation methods allowed us to recover three-fold the number of genera previously observed in regional studies aiming for the isolation of marine actinobacteria. For instance, Torres-Beltrán *et al*. (2012), using six culture media isolated 235 actinobacteria strains from Bahía de los Ángeles and Bahía Concepción marine sediments [89]. Although there was a greater number of isolated strains, they were only represented by three different genera (*Streptomyces, Micromonospora and Salinispora*), with the genus *Streptomyces* showing the highest abundance (71 %) [89]. In addition, Becerril *et al*. (2012) identified a total of 139 actinobacteria strains isolated from the Gulf of California and Todos Santos Bay marine sediments, using six culture media [90]. The genera *Salinispora* and *Streptomyces* were the most abundant genera found by Becerril (2012) with 43 and 31 %, respectively [90]. With regards to the genus *Salinispora,* we expected to recover this genus from the Laguna Ojo de Liebre sediments as there is evidence suggesting this is commonly distributed in tropical and subtropical marine sediments where its growth requirements are met [91]. However, it has also been suggested that exposure to low temperatures may have a negative effect on growth and survival of this genus [91]. Thus, it is likely that extended storage of sediment on ice for transportation to the laboratory may have had an impact on this genus recovery. Although the genus *Streptomyces* is one of the most common genera in marine sediments [35], in our study this genus only represented 3 % of actinobacteria strains isolated. Commonly *Streptomyces* strains isolated from marine environments belong to soil-inhabiting strains that have been washed from land and have adapted to the environment in marine sediments as dormant spores [92]. However, the Ojo de Liebre Lagoon has no terrestrial input, suggesting the lagoon’s environmental conditions may allow for ‘rare’ actinobacteria to flourish in the sediments. ‘Rare’ actinobacteria are those that do not belong to the genus *Streptomyces* and that have been little studied [35]. In our study, the majority of isolated actinobacteria (97 %) are affiliated with rare genera, mainly affiliated with the *Micromonospora* (41 %) and *Actinomadura* genus. The high recovery of *Micromonospora* isolates matched with recent observations in marine environments. The *Micromonospora* genus has been frequently isolated from marine environments including sea sands [93], near-shore coastal sediments [94, 95], deep-sea sediments [96, 97] and hypersaline environments [85, 86]. Phylogenomics studies have revealed that 16S rRNA is a highly conserved gene that might misclassify new species [98, 99]. The cut-off of 98.63 % for 16S rRNA species [100] have shown that species delimitation is not possible for some genera, suggesting that any nucleotide change in the 16S sequence (>99 %) could uncover new species [101–103]. Phylogenomic analysis will need to be conducted in our LOL strains in order to classify them as new species within the genus *Micromonospora* and *Actinomadura *. Schorn *et al*. (2016), evaluated the potential of rare actinomycetes and observed that genera such as *Nocardiopsis *, *Pseudonocardia* and *Actinomadura* present a high diversity of metabolic pathways with the potential to produce compounds with biological activity [104], supporting the Ojo de Liebre Lagoon may be considered as a novel source of actinobacteria with biotechnological potential.

Here we investigated the biotechnological potential of rare actinobacteria following two approaches. First, we tested for the cytotoxic activity of crude extracts obtained from actinobacteria cultures. Cytotoxicity of 11 extracts showed a cell viability decrease for colon (Caco-2), lung (H1299), liver (Hep G2) and cervical (HeLa, SiHa and CaSki) cancer cell lines, particularly crude extracts of rare actinobacteria strains affiliated with the *Micromonospora* and *Actinomadura *. Generally, most of the *Micromonospora *-derived extracts show high anticancer effects towards various human cancer cell lines, since it has been reported this actinobacterial genus commonly produce secondary metabolites belonging to the macrolides, aminoglycosides, and ansamycins class of compounds [105]. Anticancer activity for the *Micromonospora* has been previously reported for strains isolated from the Gulf of California sediments in Mexico [89, 106]. Similar to our observations, studies from Cardoso-Martinez *et al.* [106] and Torres-Beltrán *et al.* [89]*, Micromonospora* extracts showed cytotoxic activity against cervix (HeLa), colon (HCT116), lung (H460) and mama (MCF7) cancer cell lines. In addition, we observed the highest cytotoxic activity for all six cancer lines tested from *Actinomadura* crude extracts. Anticancer activity for marine *Actinomadura* has been previously reported for a myriad of cancer cell lines including colon (HCT116, CCL HT29), mama (MCF7), liver (Hep G2), melanoma (SK-Mel-147, MEXF 514L) and lung (LXFA 526L, LXFL 529L) [107] due to the genus capability of producing metabolites with strong anticancer activity, such as staurosporine and chandrananimycin A-C [108, 109]. Although, the fact we used crude extracts instead of pure compounds may raise several questions about whether the activity we observed is due to the coordinated expression of metabolites causing a potential synergistic effect or vice versa, our observations state a first screening for cytotoxic activity from rare marine actinobacteria isolated from an hypersaline environment that could lead, with support of the molecular network analysis, to future directed analysis to exactly pinpoint the chemical nature of the activity here observed.

Second, we used molecular networking as a useful tool for the analysis and visualization of complex crude extracts, and the annotation of specialized metabolites. Along with curated searches in comprehensive libraries like the Natural Products Atlas, molecular networking facilitates prioritization of metabolites based on particular interests, for example novel chemistry. From the resulting molecular network, and MS/MS data analysis we could annotate three known metabolites, including the macrolide antibiotic Rosamicin from *Micromonospora* spp. [68], the fatty amide 13-Docosenamide, and the glycoside Sarmentoside B. Rosamicin is an important broad-spectrum antibiotic with clinical efficacy. It has been observed that its activity against both Gram-positive and Gram-negative bacteria is equal or superior to erythromycin [110]. Moreover, new modifications of the antibiotic result in additional activities, for example enhanced efficacy against Methicillin-resistant *Staphylococcus aureus* (MRSA) [111, 112]. Since GNPS dereplication results can be taken as a chemical hypothesis of the metabolites produced, it would be interesting to further pursue the *Micromonospora* strains where Rosamicin was identified, for antibiotic activity and structural dereplication to characterize which member from the Rosamicin family these novel strains produce. Although not originally isolated from actinobacteria, Sarmentoside B has previously been detected in extracts from *Streptomyces cavourensis* isolated from different species of sea cucumbers [113]. Similarly, 13-Docosenamide was detected by GCMS in crude extracts from *Corynebacterium* species [114] isolated from petroleum contaminated soil, and from *Streptomyces* sp. CB-75, isolated from Banana rhizosphere soil [115].

Almost half of the nodes populating the network come from *Micromonospora* strains agreeing with the proportion of samples belonging to this group. However, we identified only one specialized metabolite from the genus. Regarding *Actinomadura* and *Nocardiopsis* strains we could not confidently match any ions to the GNPS library nor the Natural Products Atlas. The large number of unidentified ions indicate the potential strains from unexplored environments have to produce novel chemistry. Despite having low accuracy mass spectrometry data, the small number of matches to natural products libraries encourages us to investigate the specialized metabolism of these strains further; it also evidences the disproportion in chemical information available in natural product libraries from rare actinobacterial groups, since they are generally understudied. Fortunately, rare actinomycetes are receiving more recognition as the source of potent bioactive compounds, specially the genera *Actinomadura *, *Micromonospora* and *Amycolatopsis* [116]. We selected the SiHa cell viability assay results to scale the node size in the network, being the most susceptible cell line we tested; in this regard the larger nodes are those associated with the *Actinomadura* strains LOL-011 and LOL-016, which showed cytotoxic activity in most of the cell lines we tested (Fig. 3). However, we didn’t observe any known anticancer or cytotoxic compounds in our library search, either from *Actinomadura* or other taxa. These results encourage the further investigation of bacterial strains from rare taxa and unexplored environments. There is little representation of natural products from rare actinobacteria in the databases, and having isolates of rare actinomycetes from extreme environments like the Ojo de Liebre Lagoon can increase the chances of finding novel metabolites. Moreover, it could plant the seed to further research what the effects of adaptation to hypersaline environments might have over the specialized metabolism of these actinobacteria.

Our results showed taxonomic and functional information generated from marine actinobacteria isolates from the Ojo de Liebre Lagoon, Baja California Sur, México. Our observations contributed to generating fundamental and unprecedented knowledge on the culturable bacterial diversity that Ojo de Liebre harbours. In particular, we provided insight on the actinobacterial community composition and the potential role of rare actinobacteria as a novel source of bioactive compounds with pharmacological potential. Our taxonomic analyses demonstrated that the genus *Micromonospora* are the most abundant and widespread actinobacteria in the Ojo de Liebre Lagoon. We also identified temporal patterns of actinobacteria distribution as a function of sediment seasonal dynamics. Furthermore, we provided evidence on potential novel bioactive compounds being produced by rare actinobacteria strains as shown with molecular networking analysis. In addition, cytotoxic assays showed anticancer activity from crude extracts isolated from these strains, supporting actinobacteria from the Ojo de Liebre Lagoon could produce bioactive compounds with biotechnological potential.

We support the further characterization of strains such as full genome sequencing and multi-locus analysis in order to better inform about strains phylogenetic affiliation and metabolic potential. Combined, our observations provided baseline information that could be considered in future studies looking for the isolation of novel bioactive compounds with potential biotechnological use, as well as those looking for the isolation of novel actinobacterial strains for genome mining analysis which could derive the expression of metabolic routes with biotechnological potential.

## Supplementary Data

Supplementary material 1
