# Supplementary material for: Rare actinobacteria isolated from the hypersaline Ojo de Liebre Lagoon as a source of novel bioactive compounds with biotechnological potential
Source: Microbiology (Reading). 2022 Feb 25;168(2):001144. doi: 10.1099/mic.0.001144 (PMC8941997; doi:10.1099/mic.0.001144)
Supplement: Supplementary material 1 [file mic-168-1144-s001.pdf]

SUPPLEMENTARY MATERIAL

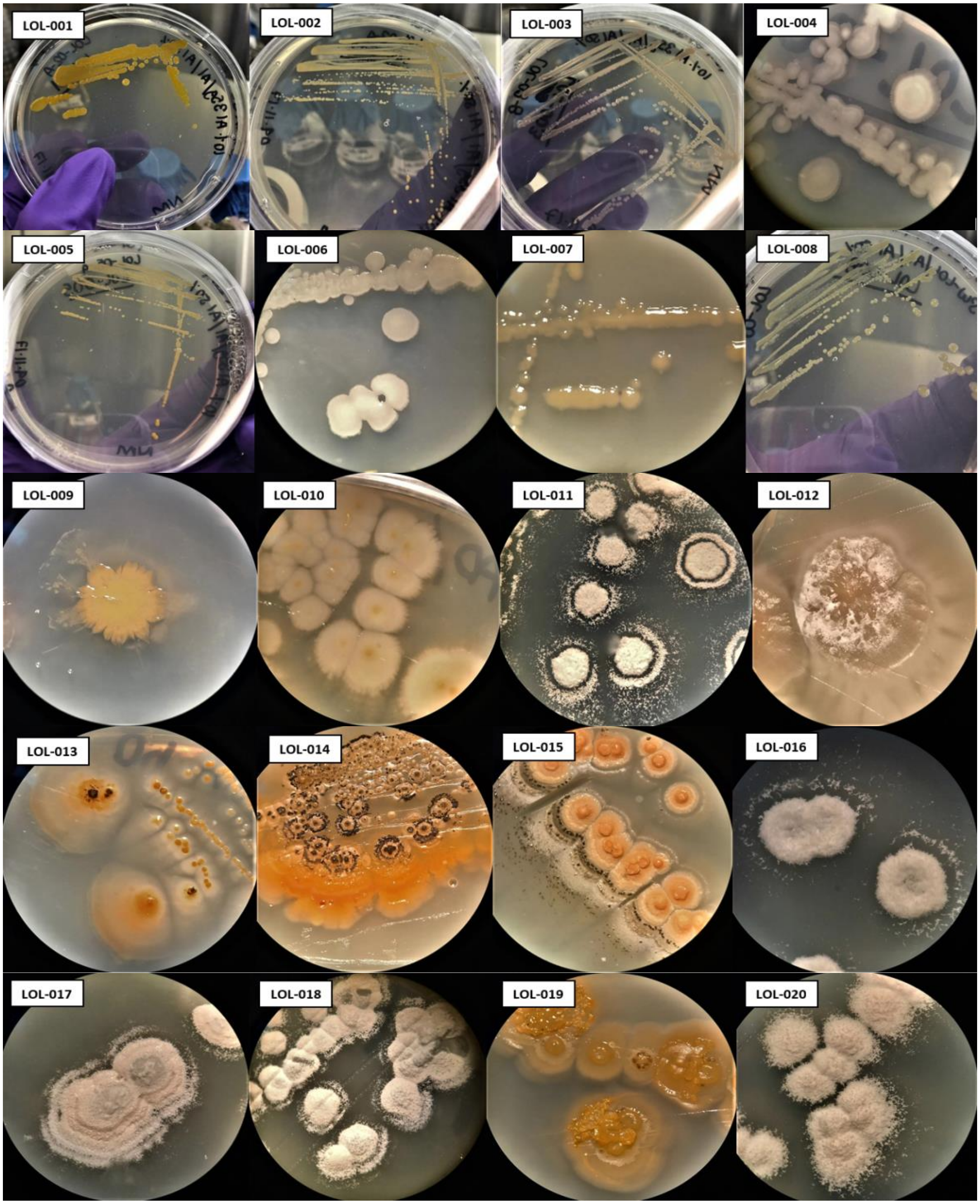

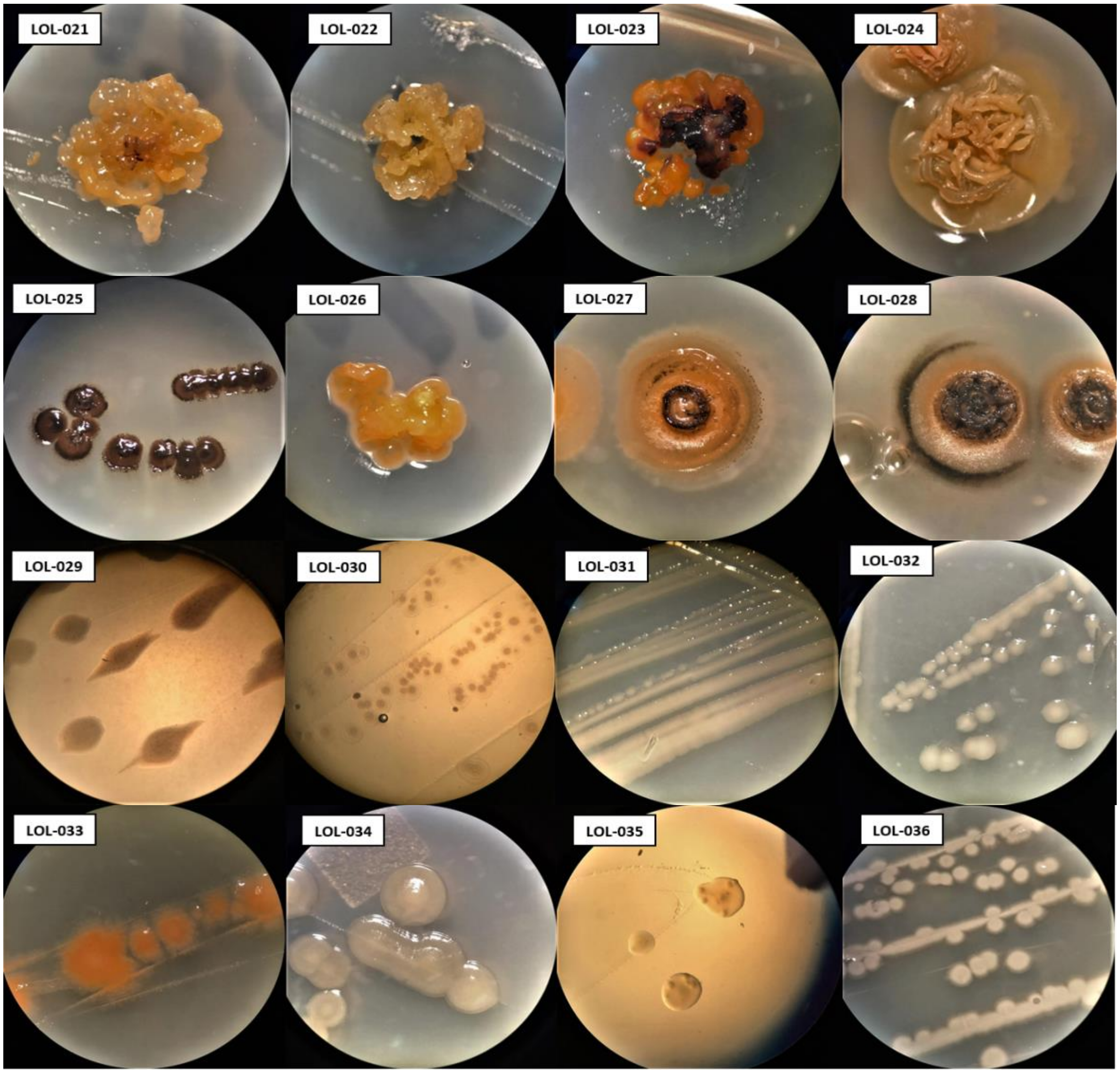

LOL-021
LOL-022
LOL-023
LOL-024
LOL-025
LOL-026
LOL-027
LOL-028
LOL-029
LOL-030
LOL-031
LOL-032
LOL-033
LOL-034
LOL-035
LOL-036

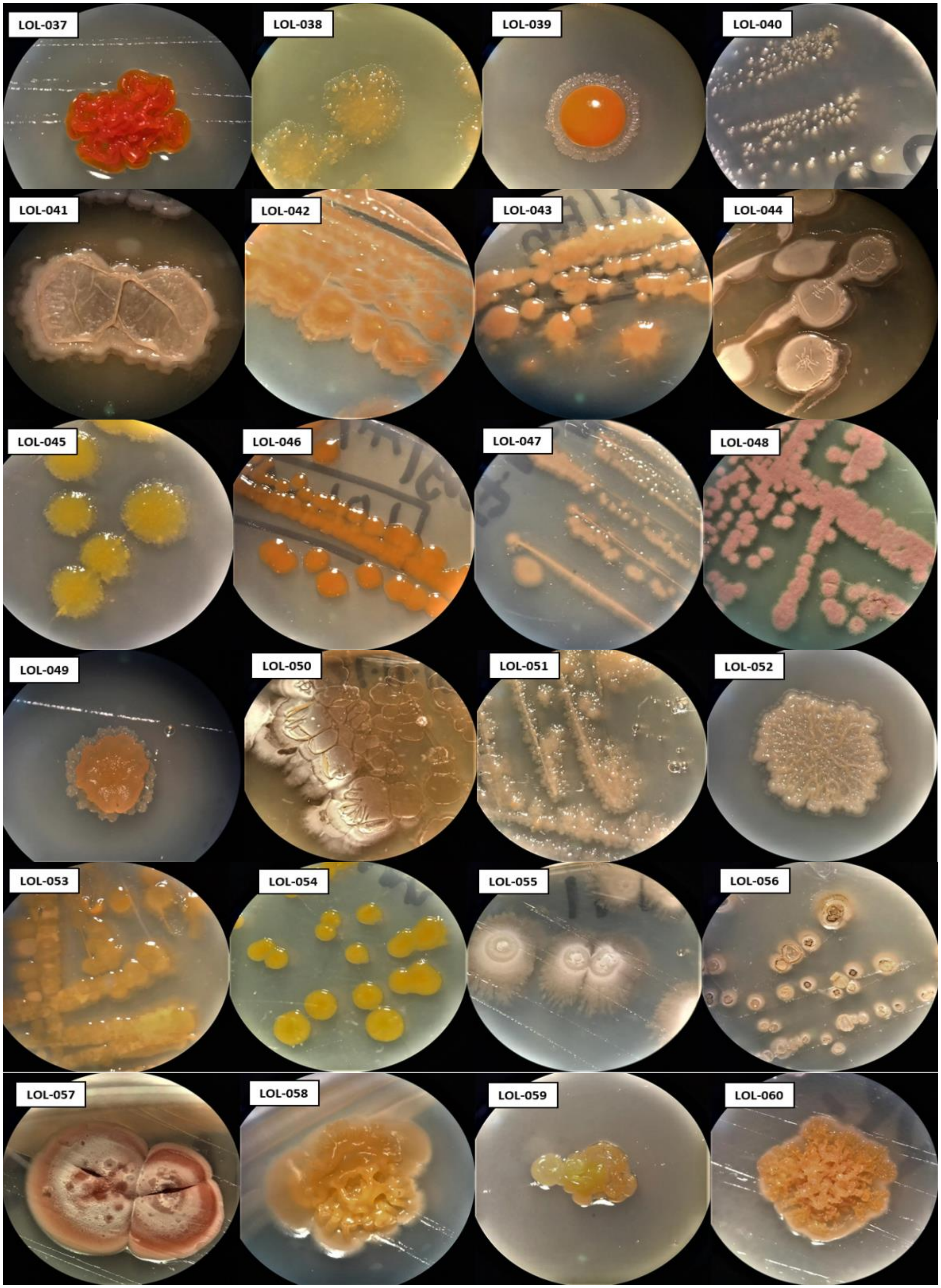
LOL-037
LOL-038
LOL-039
LOL-040
LOL-041
LOL-042
LOL-043
LOL-044
LOL-045
LOL-046
LOL-047
LOL-048
LOL-049
LOL-050
LOL-051
LOL-052
LOL-053
LOL-054
LOL-055
LOL-056
LOL-057
LOL-058
LOL-059
LOL-060

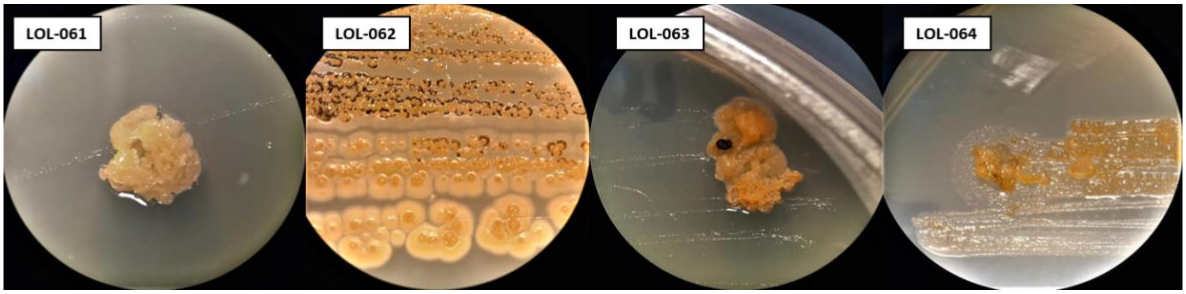

Supplementary Figure 1. Morphology of the 64 isolated strains taken with a Labomed Microscope stereomicroscope.

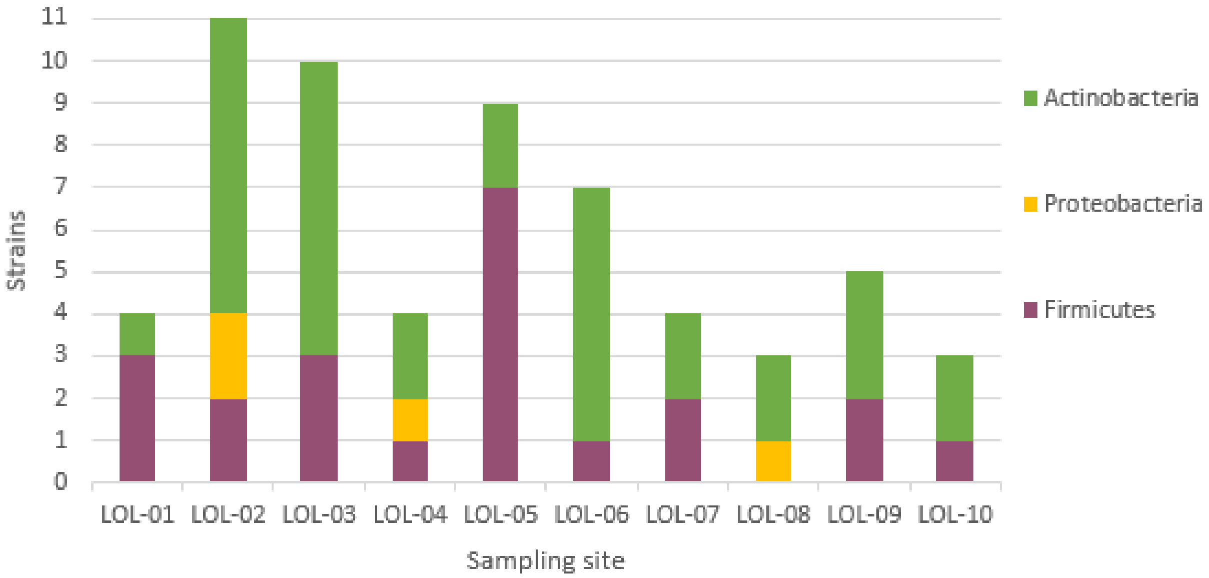

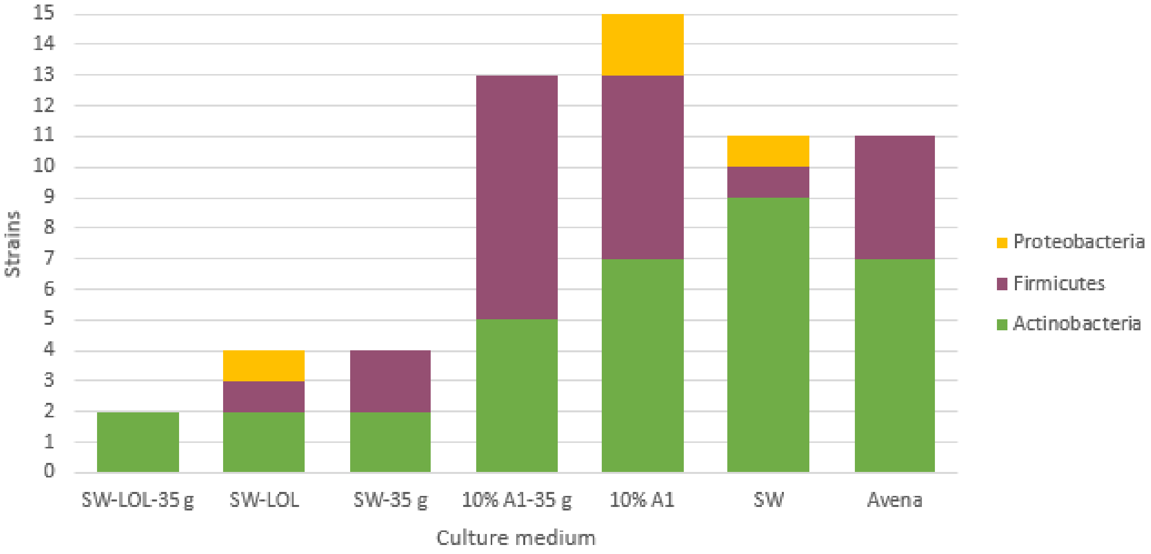

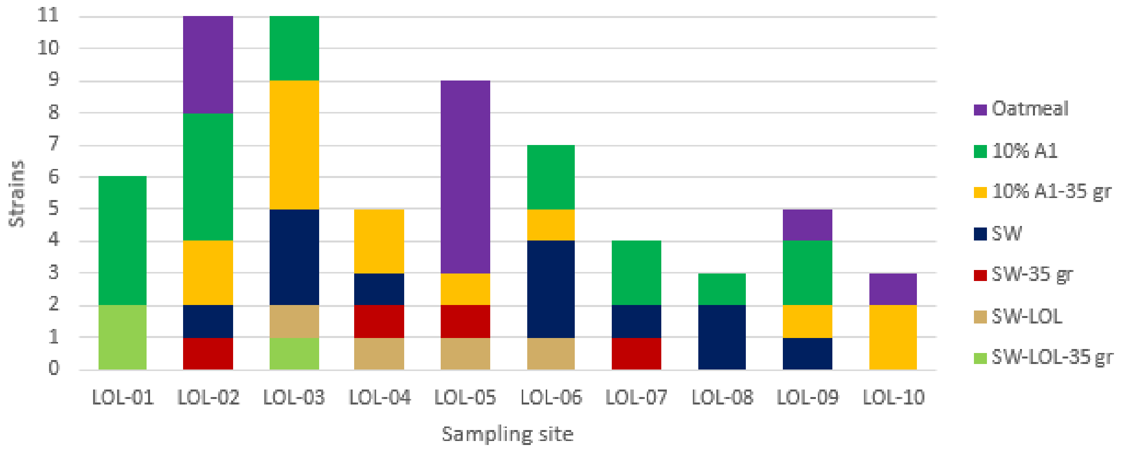

Supplementary Figure 2. A) Distribution and abundance of strains identified by sampling station. **B)** Abundance of isolated strains according to the culture medium. **C)** Distribution and abundance of the isolated strains by station based on the culture medium used.

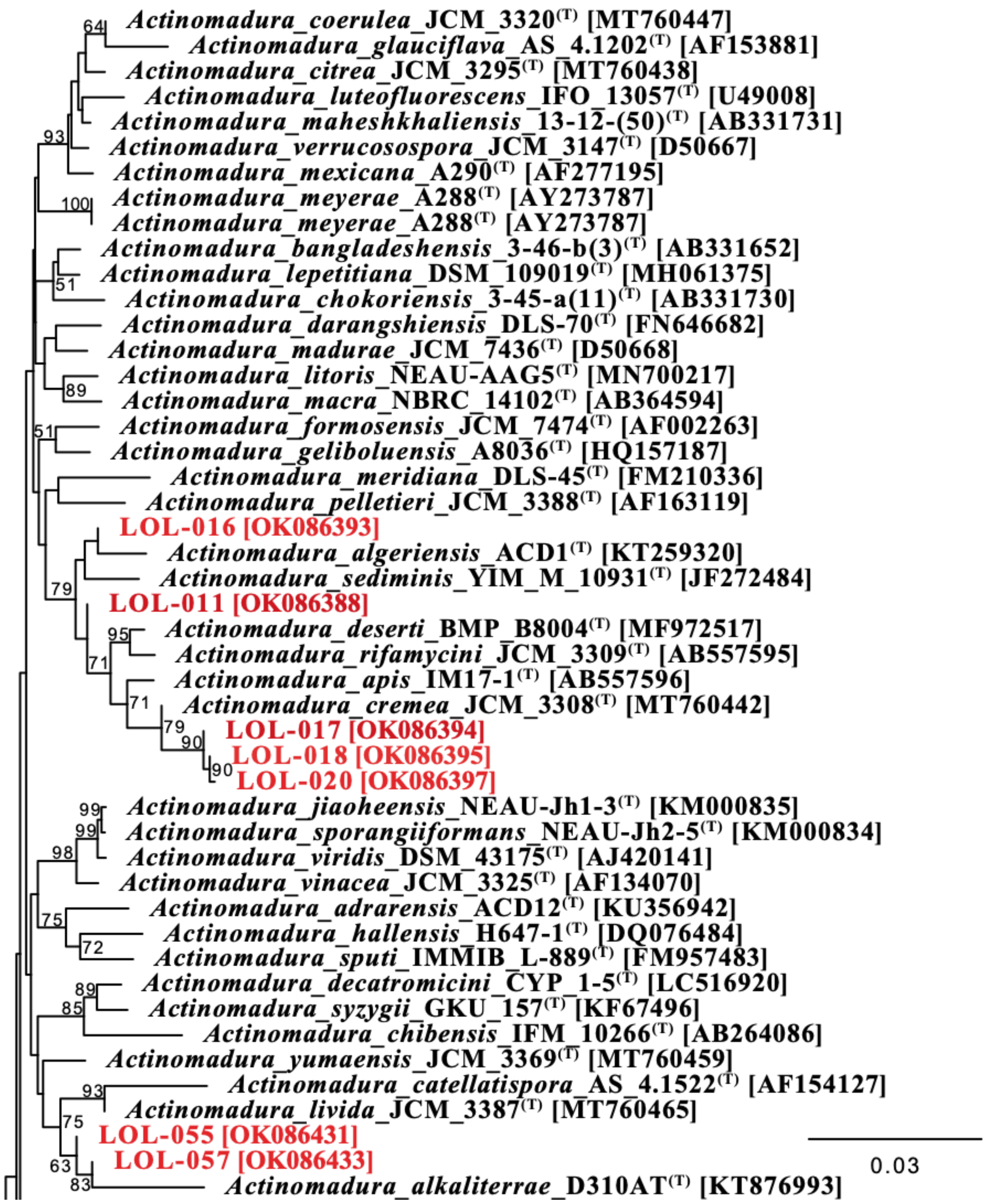

Supplementary Figure 3. Neighbor-joining tree based on 16S rRNA gene sequences of LOL-strains (red-colored) and 40 type strains (T) of described species of the genus *Actinomadura spp.* The tree includes [Accession numbers] and bootstrap values higher than 50%. Tamura-Nei model was used.

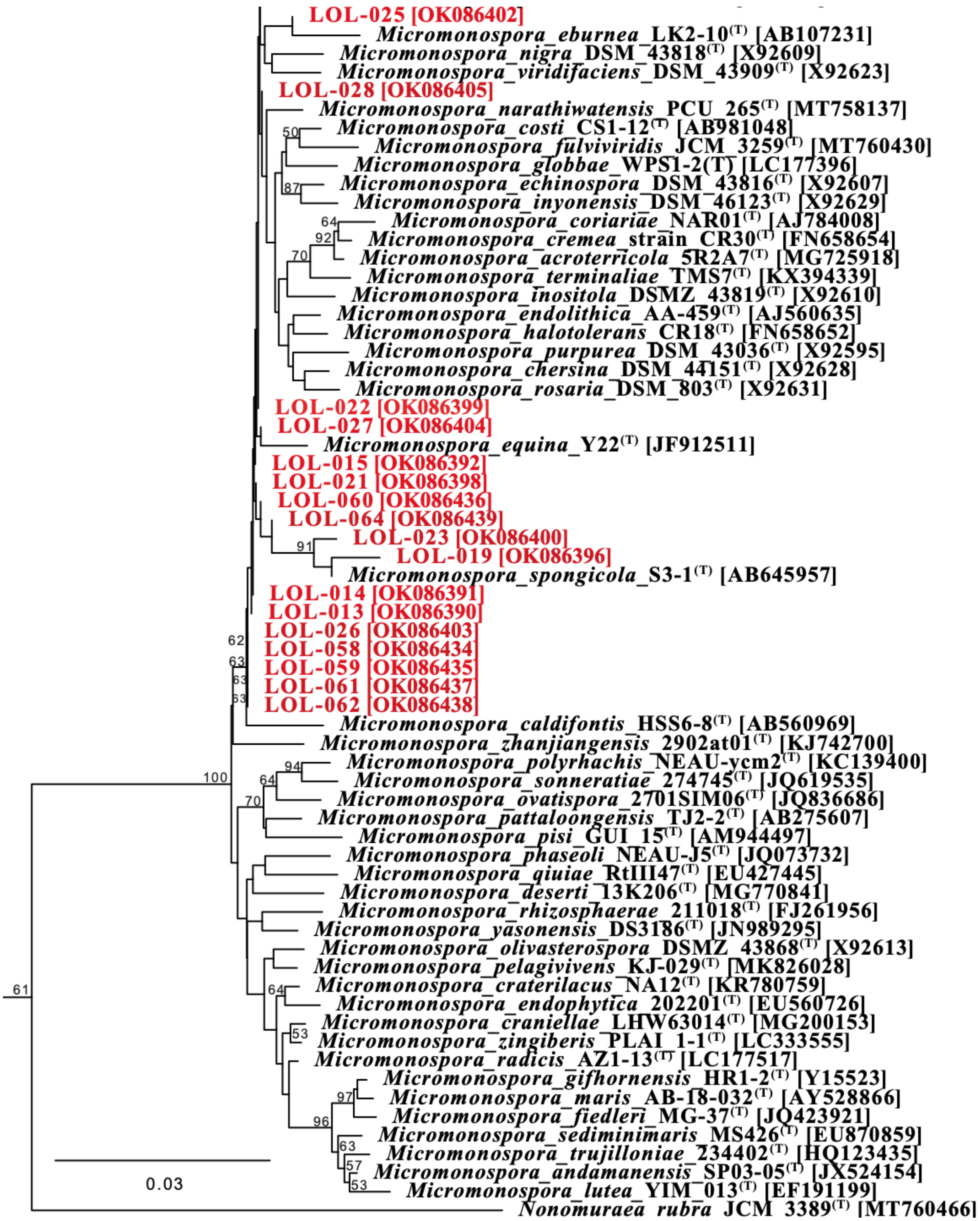

Supplementary Figure 4. Neighbor-joining tree based on 16S rRNA gene sequences of LOL-strains (red-colored) and 51 type strains (T) of the genus *Micromonospora spp.* Also includes other genera that had previously described as *Micromonospora,* including: 1T of the genus *Nonomuraea spp.* The tree includes accession numbers and bootstrap values higher than 50%. General Time Reversible model was used.

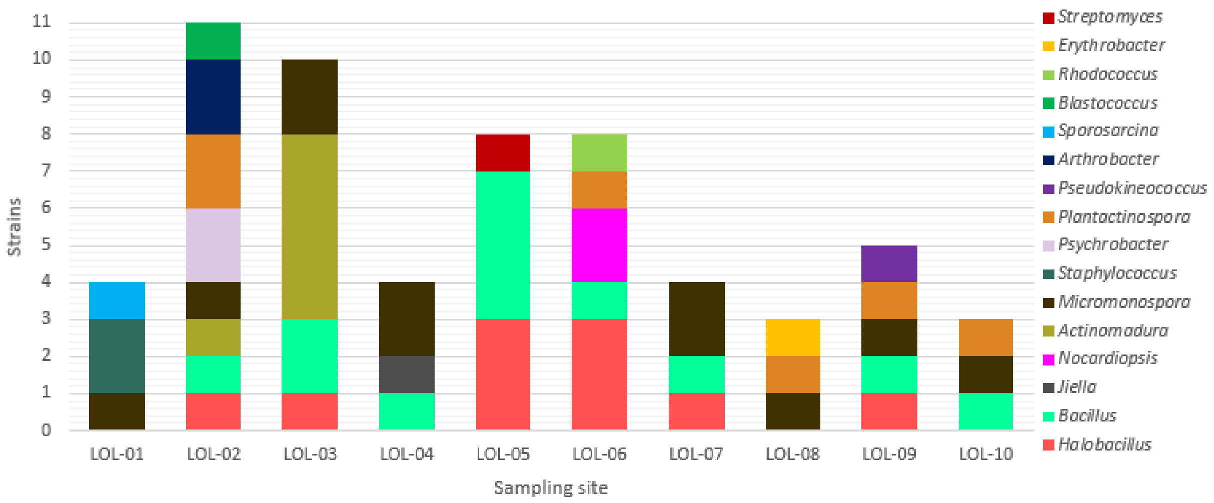

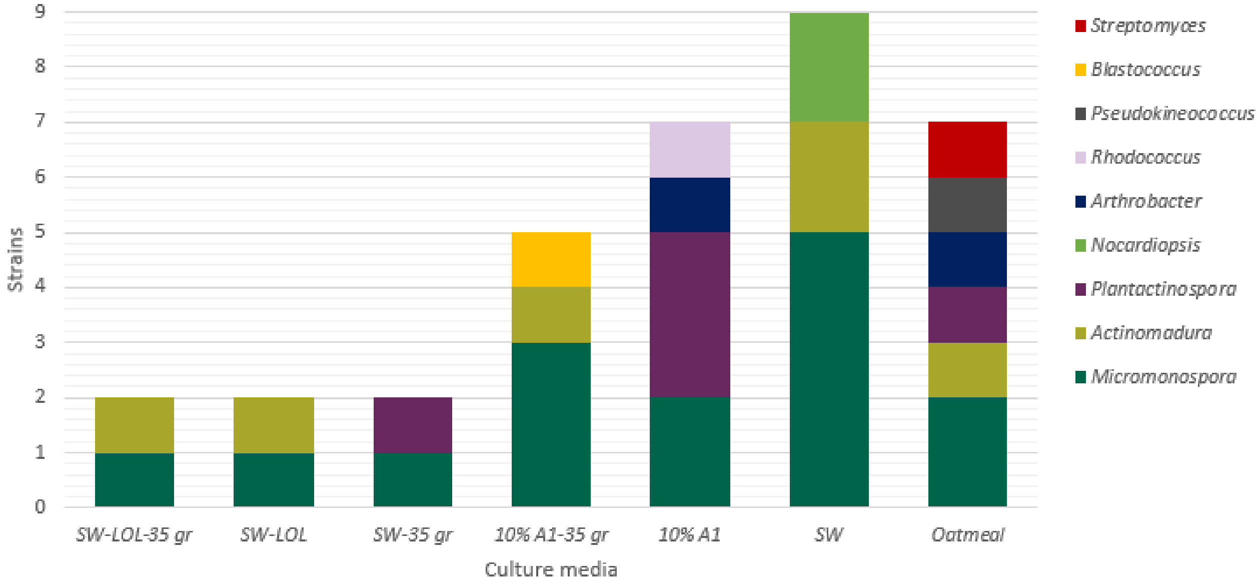

Supplementary Figure 5. A) Distribution and abundance of genera of bacterial strains by sampling site. **B)**Distribution of Actinobacteria according to the culture medium.

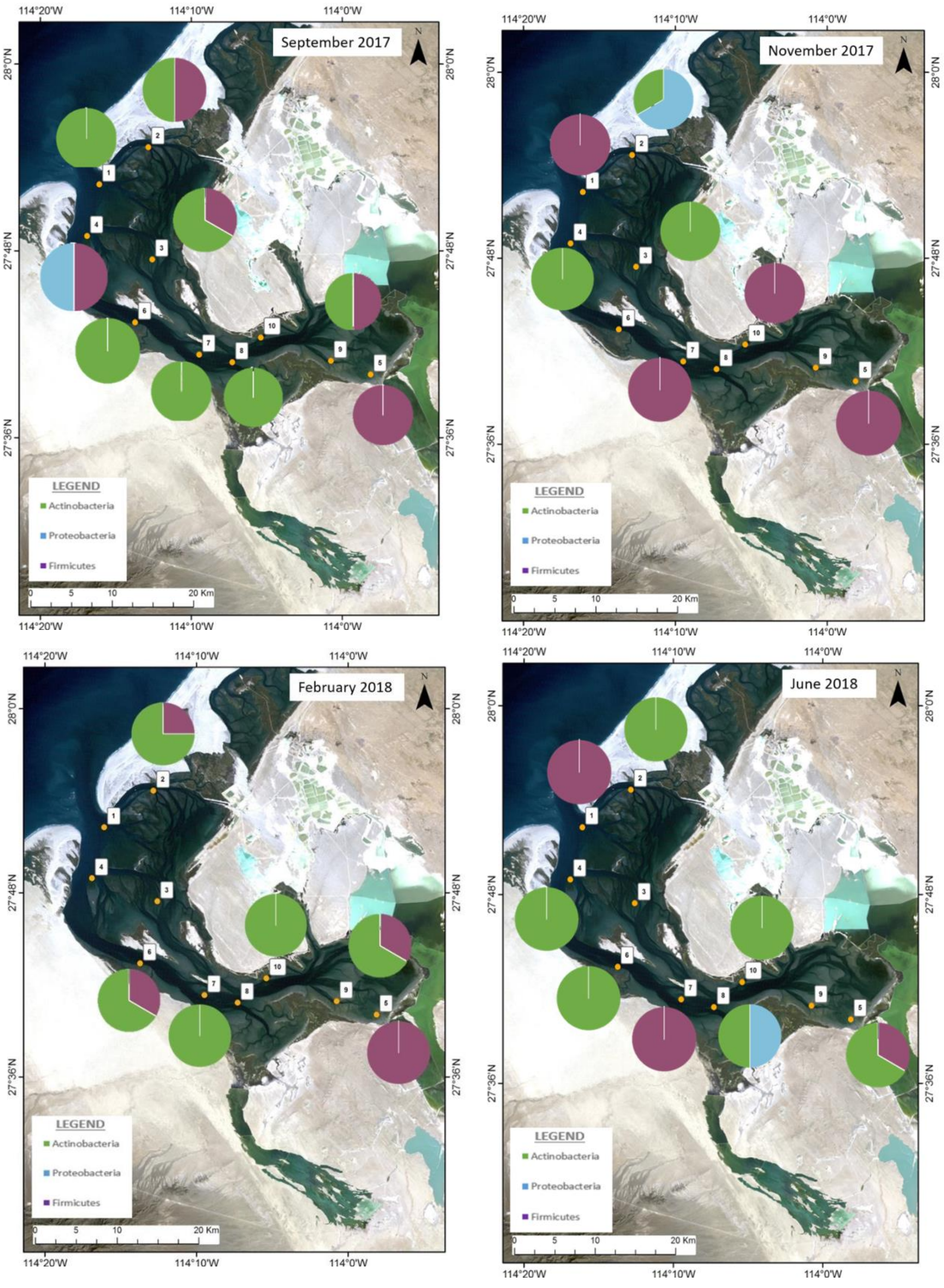
September 2017
November 2017
February 2018
June 2018
LEGEND
Actinobacteria
Proteobacteria
Firmicutes
114°20'W
114°10'W
114°0'W
28°0'N
27°48'N
27°36'N
0 5 10 20 Km
N
1
2
3
4
5
6
7
8
9
10

Supplementary Figure 6. A) Bacteria distribution map isolated from the sampling season September 2017. **B)** Distribution map of bacteria strains isolated from the sampling season November 2017.**C)** Distribution map of strains of bacteria isolated from the sampling season February 2018**. D)** Distribution map of bacteria strains isolated from the sampling season June 2018.

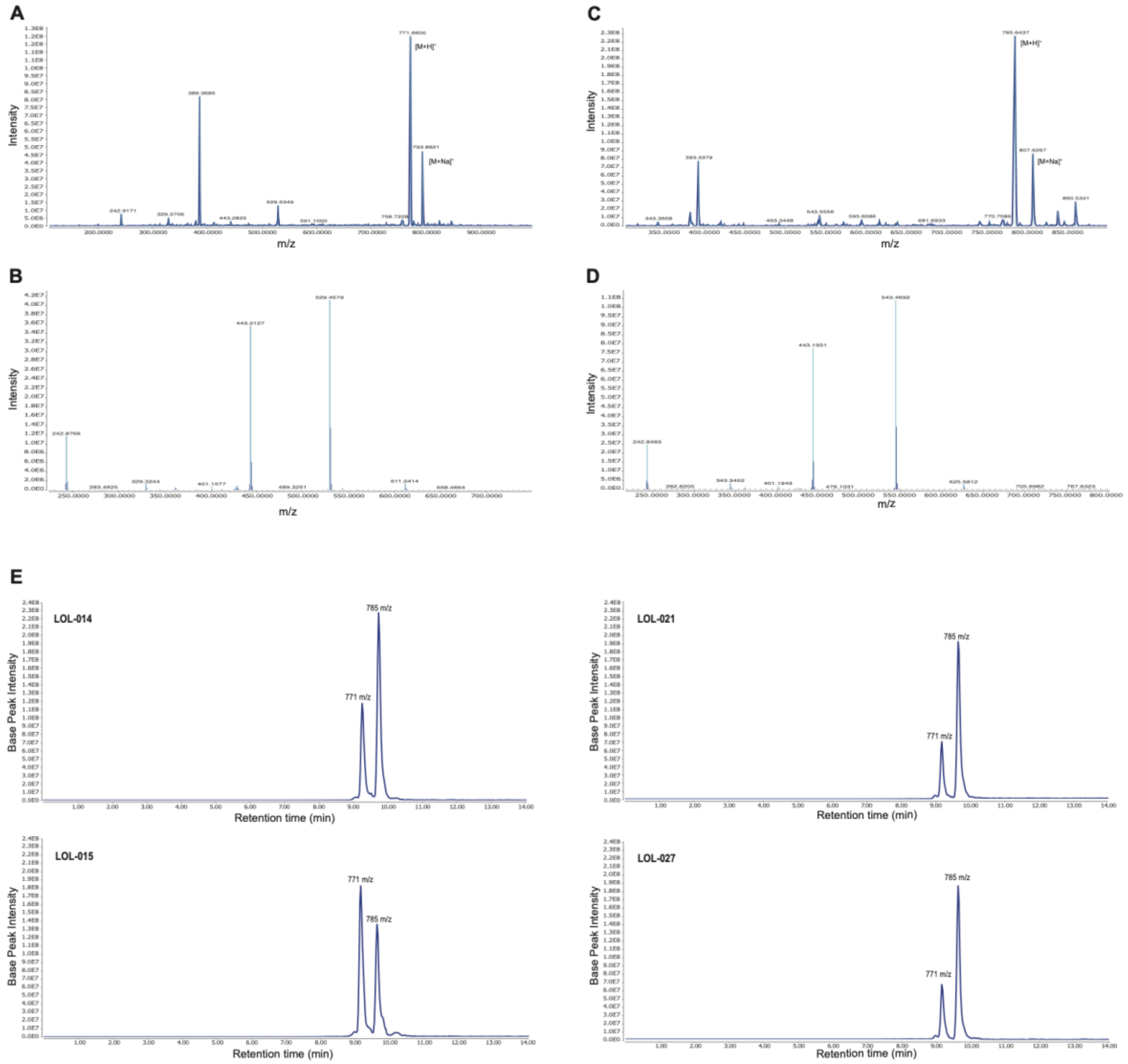

Supplementary Figure 7. LCMS data and fragmentation pattern of the 771 m/z and 785 m/z peaks. **A)** Mass spectrometry data of 771 m/z peak, indicating the putative parent ion 771 m/z ($[M+H]^+$) and the sodium adduct 793 m/z ($[M+Na]^+$). **B)** Fragmentation pattern of 771 m/z peak**. C)** Mass spectrometry data of 785 m/z peak, indicating the putative parent ion 785 m/z ($[M+H]^+$) and the sodium adduct 807m/z ($[M+Na]^+$). **D)** Fragmentation pattern of 785 m/z peak **E)** Extracted Ion Chromatogram of the *Micromonospora sp.* strains showing the 771 m/z and 785 m/z peaks; LOL-014 (top left), LOL-015 (bottom left), LOL-021 (top right), and LOL-027 (bottom right).

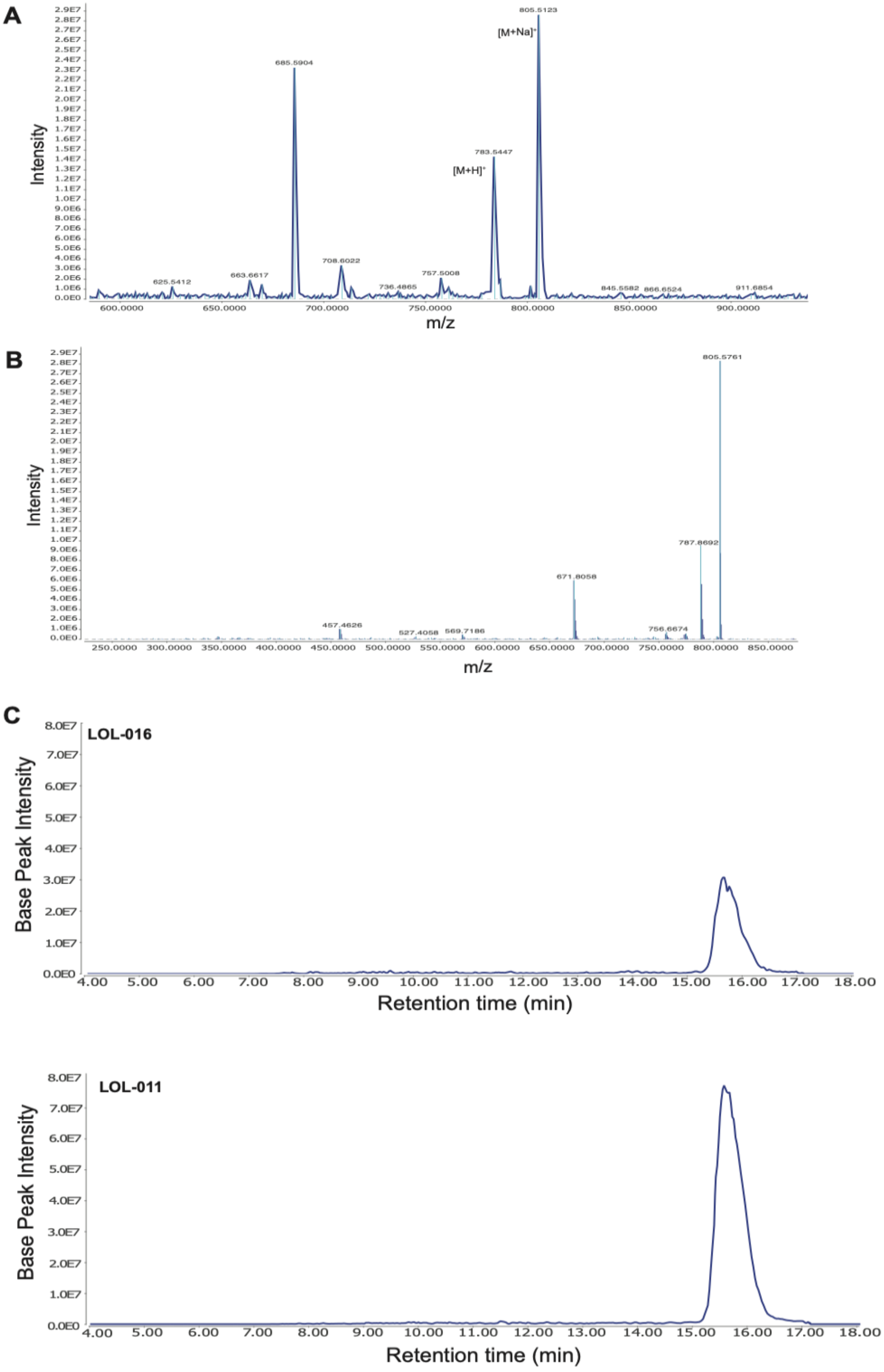
A
Intensity
m/z
805.5123
[M+Na]+
685.5904
783.5447
[M+H]+
625.5412
663.6617
708.6022
736.4865
757.5008
845.5582
866.6524
911.6854
B
Intensity
m/z
805.5761
787.8692
671.8058
457.4626
527.4058
569.7186
756.6674
C
LOL-016
Base Peak Intensity
Retention time (min)
LOL-011
Base Peak Intensity
Retention time (min)

Supplementary Figure 8. LCMS data and fragmentation pattern of the 805 m/z peak. **A.** Mass spectrometry data, indicating the putative parent ion 783 m/z ($[M+H]^+$) and the sodium adduct 805 m/z ($[M+Na]^+$) **B.** Fragmentation pattern of 805 m/z peak **C.** Extracted Ion Chromatogram of the *Actinomadura sp.* strains LOL-011 (bottom) and LOL-016 (top), showing the 805 m/z peak.

Supplementary Table I. Salinity (PSU) and temperature (ºC) of each station for each sampling season.

Sampling site	September		November		February		June	
	Temperature	Salinity	Temperature	Salinity	Temperature	Salinity	Temperature	Salinity
S1	21.8	34.5	20.5	34.6	19.38	37.8	19.47	34.4
S2	22.6	35.8	20.6	35.3	19.38	30.5	20.71	34.4
S3	22.9	30.0	20.7	35.2	20.26	39.1	21.62	35.4
S4	22.3	34.6	20.7	34.4	19.37	37.8	20.04	34.7
S5	22.5	40.0	20.1	40.0	19.42	47.1	22.35	42.8
S6	23.9	34.8	20.9	36.9	18.75	40.5	21.15	36.9
S7	23.6	37.8	20.9	36.9	19.83	41.8	21.28	37.2
S8	23.5	38.2	21.0	37.5	19.53	42.5	21.36	38.9
S9	23.2	40.0	21.0	40.0	19.4	45.3	22.04	41.5
S10	23.5	39.7	21.4	39.0	19.74	43.0	21.65	38.5

Supplementary Table II. Isolation and taxonomic data of cultured isolates and accession numbers. NI = Not Identified. Sep=September, Nov=November, Feb=February, Jun=June.

Strain number	Sampling site	Season	Culture medium	Species name	% Similarity of 16S rRNA (sequence length)	Accession number
LOL-001	S2	Sep 2017	10% A1 35 gr	*Halobacillus trueperi*	99.75 (1180 bp)	OK086378
LOL-002	S3	Sep 2017	10% A1 35 gr	*Halobacillus trueperi*	99.75 (1180 bp)	OK086379
LOL-003	S3	Sep 2017	10% A1 35 gr	*Bacillus infantis*	99.66 (1180 bp)	OK086380
LOL-004	S4	Sep 2017	10% A1 35 gr	*Bacillus rugosus*	99.82 (566 bp)	OK086381
LOL-005	S5	Sep 2017	10% A1 35 gr	*Halobacillus marinus*	99.6 (1180 bp)	OK086382
LOL-006	S9	Sep 2017	10% A1 35 gr	*Bacillus pumilus*	99.58 (1179 bp)	OK086383
LOL-007	S4	Sep 2017	SW-LOL	*Jiella aquimaris*	99.13 (1151 bp)	OK086384

LOL-008	S5	Sep 2017	SW-LOL	*Metabacillus endolithicus*	100 (677 bp)	OK086385
LOL-009	S3	Sep 2017	SW	*Alkalihalobacillus hwajinpoensis*	99.83 (1180 bp)	OK086386
LOL-010	S6	Sep 2017	SW	*Nocardiopsis fildesensis*	99.71 (684 bp)	OK086387
LOL-011	S3	Sep 2017	SW	*Actinomadura cremea*	100 (1160 bp)	OK086388
LOL-012	S6	Sep 2017	SW	*Nocardiopsis fildesensis*	99.83 (1176 bp)	OK086389
LOL-013	S8	Sep 2017	SW	*Micromonospora spongicola*	99.91 (1160 bp)	OK086390
LOL-014	S9	Sep 2017	SW	*Micromonospora spongicola*	99.91 (1160 bp)	OK086391
LOL-015	S2	Sep 2017	SW	*Micromonospora spongicola*	99.91 (1160 bp)	OK086392
LOL-016	S3	Sep 2017	SW	*Actinomadura cremea*	100 (1160 bp)	OK086393
LOL-017	S3	Sep 2017	SW-LOL 35 gr	*Actinomadura cremea*	99.85 (677 bp)	OK086394
LOL-018	S3	Sep 2017	SW-LOL	*Actinomadura cremea*	99.85 (656 bp)	OK086395
LOL-019	S3	Sep 2017	10% A1 35 gr	*Micromonospora spongicola*	100 (668 bp)	OK086396
LOL-020	S3	Sep 2017	10% A1 35 gr	*Actinomadura cremea*	99.84 (614 bp)	OK086397
LOL-021	S1	Sep 2017	SW-LOL 35 gr	*Micromonospora spongicola*	99.91 (1161 bp)	OK086398
LOL-022	S7	Sep 2017	SW	*Micromonospora spongicola*	99.91 (1160 bp)	OK086399
LOL-023	S6	Sep 2017	SW-LOL	*Micromonospora spongicola*	99.48 (1186 bp)	OK086400
LOL-024	S4	Nov 2017	SW 35 gr	*Micromonospora phaseoli*	99.91 (1160 bp)	OK086401
LOL-025	S3	Nov 2017	10% A1	*Micromonospora endolithica*	100 (1160 bp)	OK086402
LOL-026	S2	Nov 2017	Oats	*Plantactinospora endophytica*	99.86 (695 bp)	OK086403

LOL-027	S6	Feb 2018	SW	*Micromonospora spongicola*	99.91 (1160 bp)	OK086404
LOL-028	S7	Feb 2018	10% A1	*Micromonospora spongicola*	99.91 (1160 bp)	OK086405
LOL-029	S1	Nov 2017	10% A1	*Staphylococcus pasteuri*	100 (1181 bp)	OK086406
LOL-030	S1	Nov 2017	10% A1	*Staphylococcus epidermidis*	99.92 (1181 bp)	OK086407
LOL-031	S2	Nov 2017	10% A1	*Psychrobacter celer*	99.86 (720 bp)	OK086408
LOL-032	S2	Nov 2017	10% A1	*Psychrobacter nivimaris*	100 (1180 bp)	OK086409
LOL-033	S5	Nov 2017	Oats	*Cytobacillus luteolus*	99.86 (717 bp)	OK086410
LOL-034	S5	Nov 2017	Oats	*Halobacillus marinus*	99.66 (1180 bp)	OK086411
LOL-035	S7	Nov 2017	10% A1	*Halobacillus mangrovi*	99.86 (698 bp)	OK086412
LOL-036	S10	Nov 2017	10% A1 35 gr	*Bacillus zhangzhouensis*	100 (1159 bp)	OK086413
LOL-037	S9	Feb 2018	10% A1	*Micromonospora fluostatini*	99.14 (1160 bp)	OK086414
LOL-038	S9	Feb 2018	10% A1	*Halobacillus trueperi*	100 (1180 bp)	OK086415
LOL-039	S9	Feb 2018	Oats	*Pseudokineococcus marinus*	99.86 (700 bp)	OK086416
LOL-040	S6	Feb 2018	10% A1	*Plantactinospora endophytica*	99.86 (690 bp)	OK086417
LOL-041	S6	Feb 2018	10% A1 35 gr	*Bacillus nakamurai*	99.92 (1180 bp)	OK086418
LOL-042	S5	Feb 2018	Oats	*ABCF_s (Bacillus)*	99.72 (703 bp)	OK086419
LOL-043	S5	Feb 2018	Oats	*Cytobacillus luteolus*	98.51 (739 bp)	OK086420
LOL-044	S2	Feb 2018	10% A1	*Bacillus cabrialesii*	100 (1180 bp)	OK086421
LOL-045	S2	Feb 2018	Oats	*Arthrobacter subterraneus*	100 (1162 bp)	OK086422

LOL-046	S2	Feb 2018	10% A1	*Arthrobacter subterraneus*	100 (1162 bp)	OK086423
LOL-047	S1	Jun 2018	10% A1	*Sporosarcina aquimarina*	98.81 (1181 bp)	OK086424
LOL-048	S1	Jun 2018	SW	NI	NI	
LOL-049	S2	Jun 2018	10% A1 35 gr	*Blastococcus massiliensis*	99.27 (546 bp)	OK086425
LOL-050	S5	Jun 2018	Oats	*Streptomyces chumphonensis*	100 (1168 bp)	OK086426
LOL-051	S5	Jun 2018	SW 35 gr	*Halobacillus marinus*	99.66 (1180 bp)	OK086427
LOL-052	S6	Jun 2018	10% A1	*Rhodococcus nanhaiensis*	99.22 (1161 bp)	OK086428
LOL-053	S7	Jun 2018	SW 35 gr	*Alkalihalobacillus hwajinpoensis*	99.83 (1180 bp)	OK086429
LOL-054	S8	Jun 2018	SW	*Qipengyuania citrea*	100 (1154 bp)	OK086430
LOL-055	S2	Feb 2018	Oats	*Actinomadura livida*	99.40 (1169 bp)	OK086431
LOL-056	S2	Jun 2018	SW 35 gr	*Plantactinospora endophytica*	99.82 (555 bp)	OK086432
LOL-057	S3	Jun 2018	10% A1	*Actinomadura livida*	99.40 (1169 bp)	OK086433
LOL-058	S4	Jun 2018	10% A1 35 gr	*Micromonospora spongicola*	100 (505 bp)	OK086434
LOL-059	S4	Jun 2018	SW	*Micromonospora spongicola*	99.86 (732 bp)	OK086435
LOL-060	S5	Jun 2018	Oats	*Micromonospora spongicola*	99.91 (1160 bp)	OK086436
LOL-061	S8	Jun 2018	10% A1	*Plantactinospora endophytica*	99.86 (716 bp)	OK086437
LOL-062	S10	Jun 2018	Oats	*Plantactinospora endophytica*	99.86 (705 bp)	OK086438
LOL-063	S1	Jun 2018	10% A1	NI	NI	

LOL-064	S10	Feb 2018	10% A1 35 gr	*Micromonospora spongicola*	99.91 (1160 bp)	OK086439

Supplementary Table III. Cell viability (%) results for the lung (H1299), cervical (HeLa, SiHa and CaSki), colon (Caco-2), and liver (Hep G2) cancer cell lines derived from *Micromonospora* (LOL-013,14,15, 21, 23, 24, 24, 27 and 28) and *Actinomadura* (LOL-011 and LOL-016) crude extracts. Significance values are shown for each cell line (p*<0.05, p**<0.01, p***<0.001).

Strain	Cancer cell line	Cell viability (%)	Significance
**LOL-011**	CasKi	10	**
	HeLa	11	*
	SiHa	2	*
	Caco-2	18	**
	Hep G2	14	*
	H1299	8	**
**LOL-013**	CasKi	90	
	HeLa	111	*
	SiHa	70	*
	Caco-2	97	
	Hep G2	135	
	H1299	95	
**LOL-014**	CasKi	61	**
	HeLa	101	
	SiHa	75	*
	Caco-2	53	***
	Hep G2	99	
	H1299	62	
**LOL-015**	CasKi	71	*
	HeLa	92	
	SiHa	77	**

	Caco-2	85	
	Hep G2	105	
	H1299	68	
**LOL-016**	CasKi	7	**
	HeLa	8	*
	SiHa	3	***
	Caco-2	16	***
	Hep G2	18	*
	H1299	4	*
**LOL-021**	CasKi	52	**
	HeLa	82	*
	SiHa	63	*
	Caco-2	69	
	Hep G2	97	
	H1299	67	*
**LOL-023**	CasKi	62	**
	HeLa	82	*
	SiHa	89	*
	Caco-2	90	
	Hep G2	124	
	H1299	107	
**LOL-024**	CasKi	81	
	HeLa	86	*
	SiHa	84	
	Caco-2	91	
	Hep G2	105	
	H1299	72	
**LOL-025**	CasKi	63	*
	HeLa	80	*

	SiHa	43	*
	Caco-2	41	**
	Hep G2	56	*
	H1299	53	*
**LOL-027**	CasKi	48	**
	HeLa	54	*
	SiHa	34	**
	Caco-2	102	
	Hep G2	71	
	H1299	66	**
**LOL-028**	CasKi	85	
	HeLa	86	*
	SiHa	89	
	Caco-2	92	
	Hep G2	143	*
	H1299	112	
